# Comprehensive Exergy and Exergoeconomic Analyses and Optimization of a Three-Stage Cascade Refrigeration System Using Environmentally Friendly Refrigerants

**DOI:** 10.3390/e28080834

**Published:** 2026-07-23

**Authors:** Cenker Aktemur, Ezgi Gurgenc

**Affiliations:** 1 Department of Mechanical Engineering, Faculty of Engineering and Natural Sciences, Sivas University of Science and Technology, 58000 Sivas, Türkiye; 2 Department of Mechanical Engineering, Faculty of Technology, Firat University, 23119 Elazig, Türkiye; egurgenc@firat.edu.tr

**Keywords:** ultra-low temperature, global warming potential, exergoeconomic, exergy

## Abstract

Ultra-low-temperature (ULT) refrigeration systems are widely required in applications such as biomedical storage, cryogenic processing, and advanced scientific facilities, where both exergy efficiency and economic performance are critical. In this study, a comprehensive analysis and optimization of the exergy and exergoeconomic performance evaluation of a triple-stage cascade refrigeration system under ULT refrigeration is presented using ethylene (R1150), ethane (R170), propylene (R1270), difluoroethane (R152a), propane (R290), and fluoroethane (R161). A detailed exergy/exergoeconomic analysis, along with an optimization procedure, is performed at both the overall system level and the component-wise level in order to determine the key performance indicators. Minimizing the total cost product rate for each evaporator and condenser temperature is achieved by optimizing the condensing temperatures of the low-temperature cycle and the medium-temperature cycle. The component-wise analysis reveals that major thermodynamic irreversibilities occur in the HTC compressor and throttling valves, with maximum relative exergy destruction reaching 24.71% in TV-3 and the highest exergy destruction ratio reaching 14.92% in the HTC compressor. From an exergoeconomic perspective, the evaporator exhibits the largest combined exergy destruction and capital investment, and operational and maintenance cost rate (up to 24.73 $/h), while the condenser shows the highest exergoeconomic factor (up to 14.24%). The system-level results show that R1150/R170/R152a has the best exergetic and exergoeconomic performance compared to the other refrigeration combinations within the temperature ranges. Compared with R1150/R170/R290, this combination increases exergy efficiency by up to 5.05% for evaporator temperature variations and up to 7.84% for condenser temperature variations while reducing exergy destruction by up to 7.50% and 11.14%, respectively. Furthermore, the same combination decreases the total exergy destruction cost rate and product cost rate by up to 11.15% and 7.88%, respectively. In contrast, R1150/R170/R290 generally exhibits the poorest overall performance. The findings demonstrate that the refrigerant choice is crucial in achieving better exergetic and exergoeconomic performance for ULTs under various evaporator and condenser conditions.

## 1. Introduction

Refrigeration systems, which form a major part of thermodynamics, have been developing along with technology to improve the performance of refrigeration systems [1]. The concept of cascade configuration in refrigeration systems was first introduced in the 1930s to attain ultra-low-temperature (ULT) conditions ranging from −30 °C to −100 °C [2]. Single-stage vapor-compression refrigeration systems are not suitable to attain ULT conditions due to high pressure ratios [1,3,4]. Two-stage cascade refrigeration systems (CRSs) have relatively low compression ratios and higher isentropic efficiency compared to other refrigeration systems, making them superior in terms of performance [2]. Currently, two-stage CRSs are used to attain low-temperature conditions in various sectors, including the food, pharmaceutical, and chemical sectors, among others [2].

Among recent investigations (2024–2025), two-stage CRSs have predominantly focused on natural refrigerants and low-global-warming-potential (GWP) alternatives, particularly CO_2_ (R744), in the low-temperature circuit (LTC). The recent literature investigates CRSs using refrigerant pairs R744–R717 [5,6,7,8,9,10], R744–R290 [11], R744–R600 [12], R744–R134a [13], and R744–R1234yf/R1234ze(E) [14,15]. In addition, ethane-based LTCs have gained considerable attention. CRSs employing R170–R290 [16,17], R170–R600 [18], and nanoparticle-assisted R170–RE170 configurations [19] have been reported. More complex combinations, including R41/R170–R161/R290 [20] and R23–R404A [21], have also been examined for ULT applications. From the cited recent literature, the majority of the studies have focused on the investigation of the range of evaporation temperatures from −40 °C to −80 °C. Evaporation temperatures as low as −85 °C were reported by Ji et al. (2025) [17] using the R170/R290 mixture, and temperatures as low as −95 °C were reported by Kaşka and Tokgöz (2025) [20] using the R41/R170 mixture. Nevertheless, studies on temperatures below −90 °C are few, and therefore, ULT Cryogenic Refrigeration Systems below −90 °C are a developing subject of investigation.

Refrigeration techniques that are effective at temperatures below −80 °C have become increasingly important in the scientific fields of cryogenic cooling, biomedical preservation, and special freezing [22,23]. For ULT ranges below −80 °C, three-stage cascade refrigeration systems (TSCRSs) and auto-cascade refrigeration systems (TSACRSs) are employed [16,24,25,26]. Though precise ULT control is possible with the help of TSACRSs, dynamic adjustment of non-azeotropic mixtures is challenging. However, TSCRSs are more convenient for use due to their modular configuration. A TSCRS is considered to be in ideal conditions when the temperature range for evaporation is between −80 °C and −150 °C. This enables heat to be transferred at lower temperatures. This design is ideal for use in ULT refrigeration systems where single- or two-stage refrigeration may be insufficient [27].

Extensive research has been devoted to enhancing the thermodynamic performance of TSCRSs, ACRSs, and hybrid mixed-refrigerant configurations for ULT applications. As depicted in Table 1, there has been a progressive and coherent research trajectory on TSCRSs, which has covered thermodynamic performance enhancement, refrigerant transition, structural optimization, environmental evaluation, and cryogenic energy applications. Previous foundational research by Agnew and Ameli (2004) [28] focused on the finite-time thermodynamic optimization of TSCRs and the environmental and efficiency benefits of employing alternative refrigerant combinations such as R717/R508b. Further research by Sivakumar and Somasundaram (2014) [29] has provided an in-depth energy and exergy analysis of zeotropic mixtures, achieving an exergy efficiency of 58.5% at −97 °C, thereby setting an early thermodynamic milestone for TSARCSs.

Further evidence of the performance enhancement potential of hydrocarbon-based refrigerants was presented by Faruque et al. (2022b) [30]. In their study, the researchers reported a COP of 0.5931 and an exergy efficiency of 54.446% at a temperature of −100 °C. This study thus validates the thermodynamic potential of natural refrigerants. In another study, Sun et al. (2019) [27] undertook an energy and exergy analysis of a TSCRS using refrigerant substitutes with low GWP. In that study, R1150 was found to be a suitable substitute for R14 in the low-temperature stage of the TSCRS. R41 and R170 were recommended as substitutes for R23 in the medium-temperature stage. The study concluded that the substitution of refrigerants significantly affects the thermodynamic performance and environmental acceptability of the refrigerants. R170 was found to be particularly efficient and environmentally friendly. Similarly, Rogala and Kwiatkowski (2022) [31] investigated cryogenic TSCRS configurations and proved that methane (R50) is one of the rare refrigerants that can attain temperatures below 130 K in cascade compression systems.

One notable development can be observed from a series of studies by Qin et al. (2021a, 2021b, 2022a, 2022b, 2023, 2025) [32,33,34,35,36,37]. Qin et al. (2021a) [32] proposed a framework for screening refrigerants for TSACR systems, where R1234yf/R41/R14 is deemed optimal for a system operating at −100 °C. The following year, Qin et al. (2021b) [33] extended the operating range to −150 °C by employing a hybrid Linde–Hampson–ACR system with a COP of 0.1438. The development of performance optimization via intermediate pressure regulation is also seen from Qin et al. (2022a) [34], where a COP enhancement of 32.93% is realized. Qin et al. (2022b) [35] also presented a dual mixed-refrigerant scheme that reaches a temperature of −147 °C with a cooling capacity of 182.94 W. The effect of intermediate pressure on system performance is also investigated by Qin et al. (2023) [36], with experimental validation realized by Qin et al. (2025) [37] for a system operating at −124.7 °C with a stable COP value.

Regenerator-enhanced configurations further expanded the range of attainable temperatures. He et al. (2023) [38] found that three-stage regenerator-added systems increased the COP and exergy efficiency at temperatures below −100 °C. Four-stage regenerator-enhanced operation at temperatures below −140 °C was achieved by He et al. (2025) [39], who found compressors and throttling valves to be the main sources of exergy destruction.

Thermoeconomic and optimization techniques have also been increasingly employed. A recent study by Nabil et al. (2026) [40] presented a novel ejector-based TSCRS and evaluated its performance using energy, exergy, and thermo-economic analysis, along with optimization using ANN. The results show that there is a 33% enhancement in COP and a 27% improvement in exergy efficiency, along with a small increase in annual cost. In a study by Nabil et al. (2023) [26], flash tanks and suction line heat exchanger units were incorporated into the top brass refrigeration system design, resulting in a 22% improvement in COP and a 19.35% improvement in exergy efficiency. In another study, bio-inspired multi-objective optimization and 4E analysis were employed by Hamzaoui et al. (2024a, 2024b) [22,41], resulting in a COP of up to 0.9 and a total equivalent warming impact of 7.15 tCO_2_. Similarly, artificial intelligence-based parametric optimization of the system, as carried out by Pektezel (2025) [42], resulted in a COP of 0.65 at −85 °C. In a subsequent study, Pektezel and Özdemir (2026) [23] employed the response surface methodology-based multi-objective optimization approach on a TSCRS. They were able to attain a COP of 0.697 at −90 °C, along with an exergy efficiency of 41.4% and the minimization of exergy destruction. Ye et al. (2024) [43] also presented an explanation of the mixture sensitivity effect, with the COP being 0.503 and strongly dependent on the ratio of R600a to R1150.

Aside from the use of refrigeration, cascade systems have been effectively implemented for liquefied natural gas and hydrogen liquefaction plants. According to Yoon et al. (2013) [44], the COP of the cascade system for liquefied natural gas has been improved by 25%. On the other hand, Khan et al. (2016) [45] showed the simultaneous improvement of the COP and exergy efficiency for propane pre-cooling systems. Johnson et al. (2017) [46] showed the possibility of the large-scale operation of a cryogenic system at −163 °C, requiring a compression power of 28.46 MW. Pu et al. (2024) [47] optimized the design of a triple cycle for liquefied natural gas systems, resulting in the minimization of the specific power consumption. Ismail et al. (2025a, 2025b) [48,49] showed the liquefaction of hydrogen with a minimum specific energy consumption of 6.19 kWh/kg H2, resulting in a minimum of 17.4% of the total energy consumption and a significant reduction in the carbon footprint.

**Table 1:** Comparative overview of TSCRS and TSACR configurations, refrigerant selections, analysis methodologies, and key performance outcomes reported in the literature.

Reference	System Type	Refrigerants	Analysis Type	Main Outcomes
[32]	TSACR	R1234yf/R23/R14; R1234yf/R41/R14; R1234yf/R170/R14; R1234yf/R1132a/R14	Energy–Exergy	R1234yf/R41/R14 optimal for −100 °C ULT
[33]	LHR-ACR (Linde–Hampson + ACR)	R1234yf/R32; R170/R14/R50	Thermodynamic	−150 °C; COP = 0.1438; η_ex_ = 15.18%
[34]	Modified TSACR (pressure regulator)	R1234yf/R41/R14	Energy–Exergy	+32.93% COP; +15.38% η_ex_
[35]	Hybrid Linde–Hampson + TSACR	R1234yf/R32; R170/R14/R50	Experimental	−147 °C; 182.94 W; COP = 0.121
[36]	Modified TSACR	R1234yf/R170/R14	Energy–Exergy	−80 °C; +11.69% COP; +7.65% η_ex_; +15.85% $\dot{Q}$; at 1 MPa: +25.25% COP; +35.43% $\dot{Q}$
[37]	Experimental TSACR (recuperated)	R600a/R290/R170/R50	Experimental	−124.7 °C; COP = 0.1255; 93.5 W; η_carnot_ = 15.10%
[38]	3-stage TSRACR (regenerator)	R600/R170/R14	Thermodynamic	<−100 °C; +13.8% COP; +12.4% η_th_; +8.4% η_ex_
[39]	4-stage regenerator ACR	R600/R170/R14/R50	Thermodynamic	<−140 °C; COP = 0.192; η_ex_ = 0.33; major losses: compressor & throttles
[29]	TSARC	R290/R23/R14; R1270/R170/R14	Energy–Exergy	Best: R290/R23/R14; −97 °C; COP = 0.253; η_ex_ = 58.5%
[27]	TSCRS	Low-GWP substitutes	Energy–Exergy	Recommended: R1150 (LT); R41/R170 (MT); R717 (HT)
[31]	TSCRS	Various (natural & synthetic)	Energy	R50 only refrigerant cooling <130 K
[30]	TSCRS	Hydrocarbons	Thermodynamic	−100 °C; COP = 0.5931; η_ex_ = 54.446%
[41]	TSCRS	R50/R1150/R290; R50/R1150/R1270	Bio-inspired Optimization	COP up to 0.9; R290 superior
[22]	TSCRS	R1150/R170/R717; R1150/R170/R290; R1150/R170/R1270; R1150/R170/REC170	4E + Optimization	COP = 0.35–0.9; minimum TEWI = 7.15 t CO_2_ (REC170)
[26]	Modified TSCRS (flash tank + SLHX)	m-xylene/toluene/1-butene	Thermo-Economic	+22% COP; −19.33% energy; +19.35% η_ex_
[44]	Cascade LNG	C_3_H_8_/N_2_O/N_2_	Thermodynamic	+25% COP; −16% specific energy
[45]	LNG pre-cooling	Propane	Energy–Exergy	+15.51% COP; +18.76% η_ex_; −13.5% energy
[46]	Multi-stage cryogenic	CH_4_/C_2_H_4_/C_3_H_6_	Design/Optimization	−163 °C; 28.46 MW compression; 3.288 MW cooling
[48]	Triple cascade (H_2_)	MR + N_2_ loop	4E	SEC = 6.25 kWh/kg H_2_; −9% SEC; −20% CO_2_
[49]	Triple cascade (H_2_)	MR + N_2_; MR + H_2_ loop	4E	SEC = 6.98 & 6.19 kWh/kg H_2_; −17.4% & −14.5% SEC; −22% CO_2_
[47]	Triple LNG (TRC-BR)	Propane + MR	Energy–Economic	SPC = 0.270–0.273 kWh/kg; −4.84% vs. C3MR
[50]	Experimental TSACR	R600a/R1150/R50	Experimental	−125.3 °C; COP = 0.15; −90% cost; −47% CO_2_
[28]	TSCRS	R12/R13; R717/R508b	Finite-time Optimization	R717/R508b environmentally favorable
[42]	TSCRS	R1150/R170/R717	Parametric + AI	−85 °C; COP = 0.65; η_ex_ = 0.35
[23]	TSCRS	R1150/R170/R717	RSM-Based Multi-Objective Optimization	Optimal at T_evap_ = −90.043 °C; COP = 0.697; η_ex_ = 41.4%; Ė_dest_ = 5.545 kW
[43]	TSACR	R600a/R1150/R14	Thermodynamic	Best ratio 0.5/0.2/0.3; COP = 0.503; η_ex_ = 29.4%
[40]	Advanced TSCRS	1-Butene/R12/Toluene	Energy–Exergy–Thermo-Economic + ANN–GA Multi-Objective Optimization	COP ↑ 33%, Exergy efficiency ↑ 27% vs. conventional TCRS; annual cost ↑ ~5%;

Although TSCRSs have been widely investigated from a thermodynamic viewpoint, many studies have also examined them from thermo-economic, optimization and integrated 4E (energy, exergy, economics and environment) viewpoints [26,40,47,48,49,51]. However, detailed system- and component-level exergoeconomic analyses remain limited. In particular, studies that explicitly structure optimization around the Specific Exergy Costing (SPECO) methodology while accounting for different refrigerant selections at each cascade stage are absent in the existing literature. To address this, the present study proposes a comprehensive framework for the exergoeconomic evaluation and optimization of TSCRSs at the system and component levels, based explicitly on the SPECO methodology, alongside exergy analysis. The major contribution of this research effort is that a SPECO exergoeconomic approach is applied to optimize the performance of a TSCRS using alternative refrigerants for high-temperature-cycle (HTC) operation. The proposed methodology allows the refrigerant to vary in the HTC, while the low-temperature cycle (LTC) and medium-temperature cycle (MTC) are held constant. This allows the cost formation mechanisms, the distribution of exergy efficiency/destruction, and the impact of the refrigerant on the final cascade level to be systematically analyzed. Therefore, the following four refrigerant alternatives are analyzed in the HTC: R-290, R-1270, R-152a, and R-161. Conversely, R-1150 and R-170 are chosen for the LTC and MTC, respectively.

## 2. A Theoretical Model of TSCRS

### 2.1. System Description

**Figure 1:**
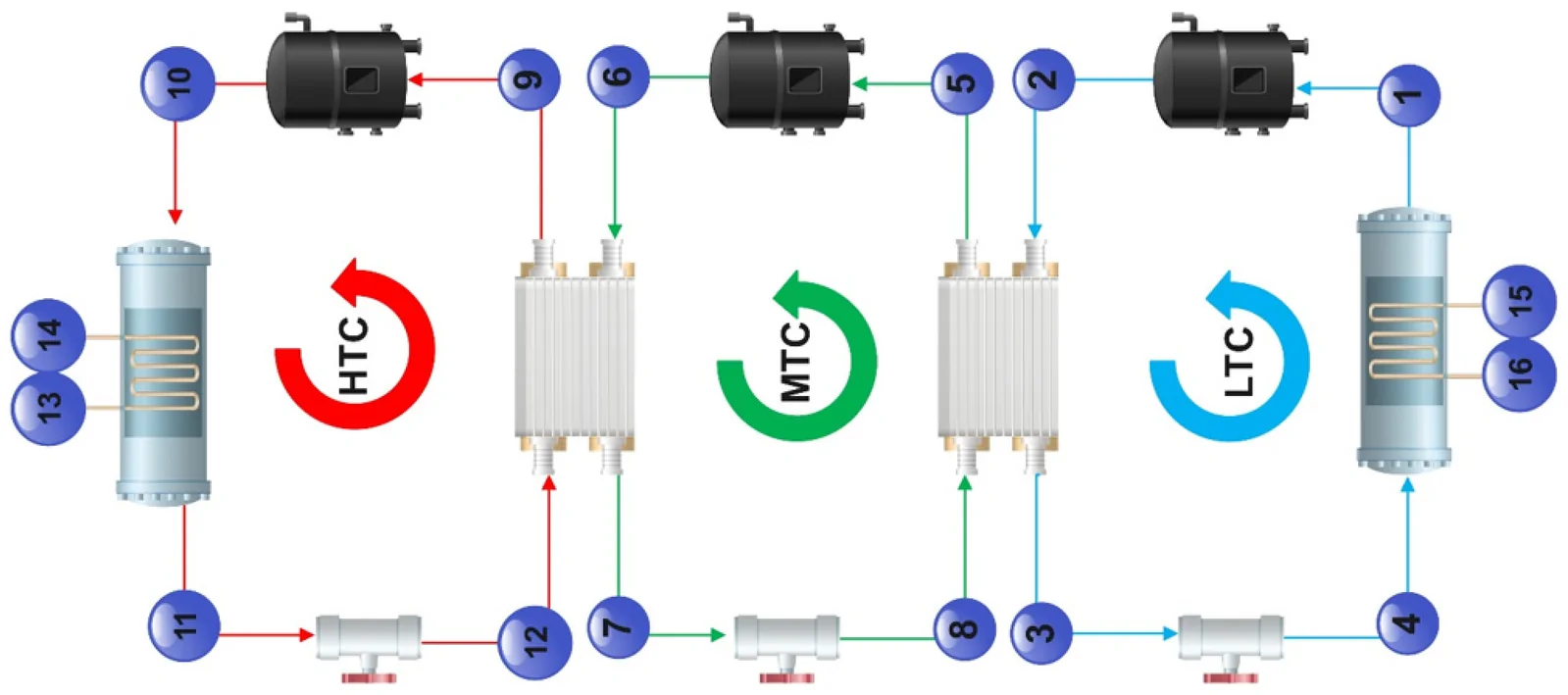
Schematic presentation of a TCRS.

The TSCRS consists of three vapor compression cycles (VCRCs) operating at different temperature levels: the LTC, MTC, and HTC, as shown in Figure 1. The LTC produces the required ultra-low refrigeration load at its evaporator. The heat rejected in the LTC condenser is not directly released to the environment; instead, it is transferred to the evaporator of the MTC through the first cascade heat exchanger (CHX-1). The MTC absorbs this heat, vaporizes its refrigerant, and subsequently rejects the absorbed heat in its condenser to the evaporator of the HTC via the second cascade heat exchanger (CHX-2). Finally, the HTC rejects the accumulated heat to the ambient environment through its condenser. In this manner, the heat is transferred stepwise from the lowest temperature level to the ambient temperature across three stages. Each cycle operates with its own compressor, expansion device, evaporator, and condenser, while the CHXs thermally couple adjacent cycles. By dividing the overall temperature lift into three smaller temperature intervals, the system significantly reduces the pressure ratio required in each compressor. This enables stable operation at ULTs, lowers discharge temperatures, improves volumetric efficiency, and enhances overall system performance compared to a single-stage configuration.

### 2.2. Refrigerant Selection

Proper selection of refrigerants can increase refrigeration potential, ensure system reliability, increase system life, and minimize environmental impacts. Under the constraints set by Regulation (EU) No 517/2014 (F-gas Regulation) and the Kigali Amendment to the Montreal Protocol, Ji et al. (2024) [16] note that refrigerants with high ODP and high GWP are being phased out. Therefore, conventional CFCs and HFCs would not be considered viable alternatives for future refrigeration technologies.

In the case of the LTC, environmentally friendly alternatives have been proposed, such as R170, R1150, R1132a, R744, and R744a. Nevertheless, the operation of the ULT system at temperatures lower than −85 °C does not allow for the attainment of the desired evaporation temperatures using R1132a and R744. Furthermore, the application of R744a would be associated with significantly low pressures, thus raising important safety and stability issues. In addition, the application of R744 would be limited to temperatures higher than −50 °C due to the presence of a triple point at −56 °C [51]. Therefore, the environmentally friendly refrigerants R170 and R1150 appear to be the most promising alternatives for the LTC [52]. In fact, R1150 has the ability to replace R14 in the LTC, resulting in a significantly reduced environmental impact. Despite the fact that both R170 and R1150 have proven to be environmentally friendly and technologically compatible with the operation of the system under ULT conditions, their flammability (A3) should be carefully assessed [52,53].

In the case of the MTC, R41 and R170 are considered to be acceptable alternatives to R23 [27] with R170 being the major alternative due to the lower environmental impact and better overall performance characteristics [22,27]. R41 has also been evaluated, but there are certain limitations and less favorable environmental characteristics compared to R170 [52].

In the case of the HTC, there are several environmentally friendly refrigerants that are commonly employed, such as R290, R717, R1270, and R1234yf, among others [16]. Among these, R717, R152a, and R161 have been specifically recommended for cascade applications [27] because of their good thermodynamic performance and relatively low GWP.

Currently, HC refrigerants employed in ULT applications are considered the most promising solutions, with the proviso that the flammability and refrigerant charge limitations are properly addressed. These refrigerants have an extremely low direct environmental impact and may be blended with nonflammable refrigerants in the upper sections of cascade systems. Recently, R1132a (A2 HFO) has been considered, although the current data are limited and mostly focus on thermophysical properties and preliminary cycle optimization studies. Overall, the shift towards low-GWP and environmentally friendly refrigerants in cascade systems requires an assessment of thermodynamic compatibility, environmental compliance, safety classification, and long-term feasibility.

The refrigerants identified in Table 2 are selected for a TSCRS in the range of −90 to −120 °C, which clearly falls in the ULT range. In the ULT range, the selection of the refrigerant is significantly constrained in terms of the LTC and MTC stages rather than being environmentally driven [27,52,53]. For the LTC, R-1150 is a technically sound choice due to its particularly low boiling point of −103.77 °C, which makes it feasible for evaporation in the −100 °C and below range without requiring excessively deep vacuum operation. R-1150’s critical temperature of 9.2 °C and critical pressure of 50.4 bar make it feasible for condensation in the cascade heat exchanger while maintaining a suitable pressure ratio. In the realm of ULT, the number of technically viable options is extremely limited. CO_2_-based refrigerants face a limitation due to their triple point, and many hydrofluoroolefin-based compounds cannot achieve the necessary ultra-low evaporation temperatures. Therefore, R-1150 is one of the few thermodynamically viable options for the LTC [23]. However, R-1150’s A3 flammability rating demands a careful approach to minimizing the charge and designing the system for safety. In the MTC, R-170 is selected because it offers an efficient thermodynamic bridge between the LTC and HTC. The normal boiling point of R-170 is −88.58 °C, while its critical temperature is 32.2 °C. R-170 is therefore efficient in matching the required temperatures within the CHX while avoiding critical compression ratios. R-170 is environmentally friendly when compared to other refrigerants such as R-23, which has a high GWP. R-170 has a GWP of 5.5, which is within the current F-gas regulations and the Kigali Amendment. Within ULTs, the choice of MTC refrigerants is further restricted by intermediate temperature requirements, which make R-170 an efficient choice [23]. In the HTC, the increased operating temperature allows for a broader choice of refrigerants. The R-290 and R-1270 refrigerants have very low values for GWP, but they fall into the A3 safety class. The R-152a refrigerant, which belongs to the A2 safety class, has lower flammability and a high critical temperature of 113.3 °C, which allows for condensation at high ambient temperatures, although it has a higher GWP of 138 compared to hydrocarbons. The R-161 refrigerant provides a good trade-off between a low GWP of 4 and good thermodynamic properties. On the whole, for a TSCRS with an operation range of −90 to −120 °C, the selection of refrigerants for the LTC and MTC sections is limited in terms of their intrinsic temperature limits. To ensure the stability and efficiency of the system, the boiling points, critical points, pressure levels, and safety ratings of the refrigerants have to be well aligned. The selected arrangement of the refrigerants (R-1150/R-170/R-290, R-1150/R-170/R-1270, R-1150/R-170/R-152a and R-1150/R-170/R-161) not only satisfies the ULT requirements but also conforms to the regulations regarding the environment while being thermodynamically viable.

### 2.3. Modeling Assumptions

Derived from the main concept of TSCRSs, a theoretical model is constructed, and in order to facilitate the analysis, certain assumptions are introduced.

(1) The pressure drop and heat loss or gain due to the flow of refrigerant inside the pipelines and other components of the system are assumed to be negligible [23,27,40,54].

(2) All the components of the system are assumed to be operating under steady-state and steady-flow conditions, with changes in kinetic and potential energies assumed to be negligible [23,27,40,54].

(3) Gas leakage is assumed to be negligible at all joints where the pipes connect to the components [27].

(4) The processes of compression and expansion are assumed to be adiabatic [23,27,54].

(5) The heat transfer is assumed to be isobaric for all heat exchange processes [54].

(6) The power consumption of the condenser and evaporator fans is not taken into consideration [54].

(7) Air is assumed to act as an external heat exchange medium in contact with the refrigerant in the condenser and evaporator [54].

(8) No subcooling is assumed at the condenser outlets, and no superheating occurs at the evaporator outlets [23,54].

### 2.4. Design Parameters and Operating Conditions

This subsection outlines the baseline parameters and operating conditions that were used to design the system. The thermodynamic model is based on these parameters and operating conditions. These include the dead-state temperature and pressure, the evaporator and condenser temperatures, and the temperature difference for the CHXs. A cooling capacity of 50 kW is also used as a baseline for comparison with previous studies and for practical purposes. Important economic parameters are also incorporated to enable an exergoeconomic analysis, including the interest rate, the plant’s lifespan, and the cost of electricity. These parameters and working conditions are based on the literature and are feasible to implement, as shown in Table 3.

### 2.5. Thermodynamic and Exergoeconomic Modeling

Energy and mass conservation equations are applied to the components under the assumption of a steady-state process to obtain the thermodynamic properties, work done by the compressor, heat transfer rates, and the performance parameters of the system, such as the COP. For the purpose of exergy analysis, the physical exergy is calculated for the state points by taking into consideration the environmental conditions. Exergy destruction is calculated for the components of the TSCRS. Energy and exergy equations for the presented model are listed in Table 4. For the purpose of exergoeconomic analysis, the SPECO method is used. In the first step of the SPECO method, the cost balance equations for the components of the TSCRS are formulated. In the process, the exergy is equated to the capital investment and operational costs. As a result, the unit cost of the product streams and the cost rate of the exergy destruction streams are calculated for the TSCRS.

The principle of conservation of mass [40,54]:

(1) $$\sum_{i}\dot{m}=\sum_{o}\dot{m}$$

The principle of conservation of energy [40,54,55]:

(2) $$\dot{Q}-\dot{W}+\sum_{i}\dot{m}h-\sum_{o}\dot{m}h=0$$

The total exergy of a stream [40,54]:

(3) $${\dot{E}}_{tot}={\dot{m}}_{i}\left[(h_{i}-h_{0})-T_{0}\left(s_{i}-s_{0}\right)\right]$$

The exergy balance for the kth component [40,54]:

(4) $$\sum_{i}{\dot{E}}_{k}=\sum_{o}{\dot{E}}_{k}+{\dot{E}}_{D,k}$$

In this study, the concepts of product, fuel and loss exergy are adopted [56,57,58]:

(5) $${\dot{E}}_{D,k}={\dot{E}}_{F,k}-{\dot{E}}_{P,k}$$

The exergy destruction ratio and relative exergy destruction ratio (or fuel depletion ratio) for each component [59]:

(6) $$y_{k}=\frac{{\dot{E}}_{D,k}}{{\dot{E}}_{F,sys}}$$

(7) $$y_{k}^{*}=\frac{{\dot{E}}_{D,k}}{{\dot{E}}_{D,sys}}$$

Total amount of destroyed exergy that occurs in the whole [56,57,58]:

(8) $${\dot{E}}_{D,sys}={\dot{E}}_{F,sys}-{\dot{E}}_{P,sys}-{\dot{E}}_{L,sys}$$

The exergy efficiency for the kth component [56,57,58]:

(9) $$\varepsilon_{k}=\frac{{\dot{E}}_{P,k}}{{\dot{E}}_{F,k}}=1-\frac{{\dot{E}}_{D,k}}{{\dot{E}}_{F,k}}$$

The general heat transfer equations of the heat exchangers [54]:

(10) $$\dot{Q}=U\times A\times LMTD$$

(11) $$LMTD=\frac{\left(T_{hot,in}-T_{cold,o}\right)-\left(T_{hot,o}-T_{hot,in}\right)}{ln\left(\frac{T_{hot,in}-T_{cold,o}}{T_{hot,o}-T_{cold,in}}\right)}$$

The cost balance equation [60]:

(12) $$\sum_{o}{\dot{C}}_{o,k}+{\dot{C}}_{w,k}={\dot{C}}_{q,k}+\sum_{in}{\dot{C}}_{in,k}+{\dot{Z}}_{k}$$

The cost flows associated with exergy flows [60]:

(13) $${\dot{C}}_{in}=c_{in}{\dot{E}}_{in}=c_{in}{\dot{m}}_{in}e_{in}$$

(14) $${\dot{C}}_{o}=c_{o}{\dot{E}}_{o}=c_{o}{\dot{m}}_{o}e_{o}$$

(15) $${\dot{C}}_{w}=c_{w}{\dot{W}}_{in}$$

(16) $${\dot{C}}_{q}=c_{q}{\dot{E}}_{q}$$

The capital recovery factor (CRF) is used to annualize the total investment cost of each component and is defined as [16]:

(17) $$CRF=\frac{{i\left(1+i\right)}^{n}}{{\left(1+i\right)}^{n}-1}$$

To update the reference purchase cost to the base year (2025), the Chemical Engineering Plant Cost Index (CEPCI) is employed [14]:

(18) $$Z_{k,2025}=Z_{k,ref}\times \frac{{CEPCI}_{2025}}{{CEPCI}_{ref}}$$

The total levelized cost rate of component k consists of capital investment (CI) and operation and maintenance (O&M) costs [59]:

(19) $${\dot{Z}}_{k,2025}^{CI+O\&M}=\frac{CRF\times {\phi \times Z}_{k,2025}}{t}$$

The unit exergy cost of the fuel stream for component k [60]:

(20) $$c_{F,k}=\frac{{\dot{C}}_{F,k}}{{\dot{E}}_{F,k}}$$

The unit exergy cost of the product stream for component k [60]:

(21) $$c_{P,k}=\frac{{\dot{C}}_{P,k}}{{\dot{E}}_{P,k}}$$

The cost rate associated with exergy destruction for component k [60]:

(22) $${\dot{C}}_{D,k}=c_{F,k}{\dot{E}}_{D,k}$$

The cost rate of exergy losses for component k [60]:

(23) $${\dot{C}}_{L,k}=c_{F,k}{\dot{E}}_{L,k}$$

The exergoeconomic factor of the kth component shows how much the cost of buying the equipment contributes to the overall cost of both the equipment and energy loss. The exergoeconomic factor for component k is [58]:

(24) $$f_{k}=\frac{{\dot{Z}}_{k,2025}}{{{\dot{Z}}_{k,2025}+c}_{F,k}\left({\dot{E}}_{D,k}+{\dot{E}}_{L,k}\right)}$$

In SPECO optimization, the objective is the minimization of the total product cost rate of the system, given by [58]:

(25) $$Min{\dot{C}}_{P,sys}=\sum_{k=1}^{N}\left({\dot{Z}}_{k}+{\dot{C}}_{F,k}\right)$$

**Table 4:** Energy and exergy equations for the presented model [54,57].

Components	Mass Balance	Energy Equations	Exergy Equations			
			Exergy of Fuel (${\dot{E}}_{F,k}$)	Exergy of Product (${\dot{E}}_{P,k}$)	Exergy Destruction Rate (${\dot{E}}_{D,k}$)	Exergy Efficiency ($\varepsilon_{k}$)
LTC compressor	${\dot{m}}_{LTC}={\dot{m}}_{1}={\dot{m}}_{2}$	${\dot{W}}_{LTC}=\frac{{\dot{m}}_{LTC}\left(h_{2s}-h_{1}\right)}{\eta_{isen}\eta_{mech}\eta_{elect}}$	${\dot{E}}_{F,LTC,comp}={\dot{W}}_{LTC,comp}$	${\dot{E}}_{P,LTC,comp}={\dot{m}}_{LTC}\left(e_{2}-e_{1}\right)$	${\dot{E}}_{D,LTC,comp}={\dot{E}}_{F,LTC,comp}-{\dot{E}}_{P,LTC,comp}$	$\varepsilon_{LTC,comp}=\frac{{\dot{E}}_{P,LTC,comp}}{{\dot{E}}_{F,LTC,comp}}$
MTC compressor	${\dot{m}}_{MTC}={\dot{m}}_{5}={\dot{m}}_{6}$	${\dot{W}}_{MTC}=\frac{{\dot{m}}_{MTC}\left(h_{6s}-h_{5}\right)}{\eta_{isen}\eta_{mech}\eta_{elect}}$	${\dot{E}}_{F,MTC,comp}={\dot{W}}_{MTC,comp}$	${\dot{E}}_{P,MTC,comp}={\dot{m}}_{LTC}\left(e_{6}-e_{5}\right)$	${\dot{E}}_{D,MTC,comp}={\dot{E}}_{F,MTC,comp}-{\dot{E}}_{P,MTC,comp}$	$\varepsilon_{MTC,comp}=\frac{{\dot{E}}_{P,MTC,comp}}{{\dot{E}}_{F,MTC,comp}}$
HTC compressor	${\dot{m}}_{HTC}={\dot{m}}_{9}={\dot{m}}_{10}$	${\dot{W}}_{HTC}=\frac{{\dot{m}}_{HTC}\left(h_{10s}-h_{9}\right)}{\eta_{isen}\eta_{mech}\eta_{elect}}$	${\dot{E}}_{F,HTC,comp}={\dot{W}}_{HTC,comp}$	${\dot{E}}_{P,HTC,comp}={\dot{m}}_{HTC}\left(e_{10}-e_{9}\right)$	${\dot{E}}_{D,HTC,comp}={\dot{E}}_{F,HTC,comp}-{\dot{E}}_{P,HTC,comp}$	$\varepsilon_{HTC,comp}=\frac{{\dot{E}}_{P,HTC,comp}}{{\dot{E}}_{F,HTC,comp}}$
LTC expansion valve	${\dot{m}}_{LTC}={\dot{m}}_{3}={\dot{m}}_{4}$	$h_{3}=h_{4}$	${\dot{E}}_{F,LTC,TV}={\dot{m}}_{LTC}e_{3}$	${\dot{E}}_{P,LTC,TV}={\dot{m}}_{LTC}e_{4}$	${\dot{E}}_{D,LTC,TV}={\dot{E}}_{F,LTC,TV}-{\dot{E}}_{P,LTC,TV}$	$\varepsilon_{LTC,TV}=\frac{{\dot{E}}_{P,LTC,TV}}{{\dot{E}}_{F,LTC,TV}}$
MTC expansion valve	${\dot{m}}_{MTC}={\dot{m}}_{7}={\dot{m}}_{8}$	$h_{7}=h_{8}$	${\dot{E}}_{F,MTC,TV}={\dot{m}}_{MTC}e_{7}$	${\dot{E}}_{P,MTC,TV}={\dot{m}}_{MTC}e_{8}$	${\dot{E}}_{D,MTC,TV}={\dot{E}}_{F,MTC,TV}-{\dot{E}}_{P,MTC,TV}$	$\varepsilon_{MTC,TV}=\frac{{\dot{E}}_{P,MTC,TV}}{{\dot{E}}_{F,MTC,TV}}$
HTC expansion valve	${\dot{m}}_{HTC}={\dot{m}}_{11}={\dot{m}}_{12}$	$h_{11}=h_{12}$	${\dot{E}}_{F,MTC,TV}={\dot{m}}_{HTC}e_{11}$	${\dot{E}}_{P,MTC,TV}={\dot{m}}_{HTC}e_{12}$	${\dot{E}}_{D,HTC,TV}={\dot{E}}_{F,HTC,TV}-{\dot{E}}_{P,HTC,TV}$	$\varepsilon_{HTC,TV}=\frac{{\dot{E}}_{P,HTC,TV}}{{\dot{E}}_{F,HTC,TV}}$
CHX-1	${\dot{m}}_{LTC}={\dot{m}}_{2}={\dot{m}}_{3}$ ${\dot{m}}_{MTC}={\dot{m}}_{5}={\dot{m}}_{8}$	${\dot{Q}}_{CHX\text{-}1}={\dot{m}}_{LTC}\left(h_{2}-h_{3}\right)$ ${\dot{Q}}_{CHX\text{-}1}={\dot{m}}_{MTC}\left(h_{5}-h_{8}\right)$	${\dot{E}}_{F,CHX\text{-}1}={\dot{m}}_{LTC}\left(e_{2}-e_{5}\right)$	${\dot{E}}_{P,CHX\text{-}1}={\dot{m}}_{LTC}\left(e_{3}-e_{8}\right)$	${\dot{E}}_{D,CHX\text{-}1}={\dot{E}}_{F,CHX\text{-}1}-{\dot{E}}_{P,CHX\text{-}1}$	$\varepsilon_{CHX\text{-}1}=\frac{{\dot{E}}_{P,CHX\text{-}1}}{{\dot{E}}_{F,CHX\text{-}1}}$
CHX-2	${\dot{m}}_{MTC}={\dot{m}}_{6}={\dot{m}}_{7}$ ${\dot{m}}_{HTC}={\dot{m}}_{9}={\dot{m}}_{12}$	${\dot{Q}}_{CHX\text{-}2}={\dot{m}}_{MTC}\left(h_{6}-h_{7}\right)$ ${\dot{Q}}_{CHX\text{-}2}={\dot{m}}_{HTC}\left(h_{9}-h_{12}\right)$	${\dot{E}}_{F,CHX\text{-}2}={\dot{m}}_{MTC}\left(e_{6}-e_{9}\right)$	${\dot{E}}_{P,CHX\text{-}2}={\dot{m}}_{MTC}\left(e_{7}-e_{12}\right)$	${\dot{E}}_{D,CHX\text{-}2}={\dot{E}}_{F,CHX\text{-}2}-{\dot{E}}_{P,CHX\text{-}2}$	$\varepsilon_{CHX\text{-}2}=\frac{{\dot{E}}_{P,CHX\text{-}2}}{{\dot{E}}_{F,CHX\text{-}2}}$
Evaporator	${\dot{m}}_{LTC}={\dot{m}}_{1}={\dot{m}}_{4}$ ${\dot{m}}_{air,evap}={\dot{m}}_{13}={\dot{m}}_{14}$	${\dot{Q}}_{evap}={\dot{m}}_{LTC}\left(h_{1}-h_{4}\right)$ ${\dot{Q}}_{evap}={\dot{m}}_{air,evap}\left(h_{13}-h_{14}\right)$	${\dot{E}}_{F,evap}={\dot{m}}_{LTC}\left(e_{4}-e_{1}\right)$	${\dot{E}}_{P,evap}={\dot{m}}_{LTC}\left(e_{14}-e_{13}\right)$	${\dot{E}}_{D,evap}={\dot{E}}_{F,evap}-{\dot{E}}_{P,evap}$	$\varepsilon_{evap}=\frac{{\dot{E}}_{P,evap}}{{\dot{E}}_{F,evap}}$
Condenser	${\dot{m}}_{HTC}={\dot{m}}_{10}={\dot{m}}_{11}$ ${\dot{m}}_{air,cond}={\dot{m}}_{15}={\dot{m}}_{16}$	${\dot{Q}}_{cond}={\dot{m}}_{HTC}\left(h_{10}-h_{11}\right)$ ${\dot{Q}}_{cond}={\dot{m}}_{air,evap}\left(h_{16}-h_{15}\right)$	${\dot{E}}_{F,cond}={\dot{m}}_{HTC}\left(e_{10}-e_{11}\right)$	${\dot{E}}_{P,cond}={\dot{m}}_{HTC}\left(e_{16}-e_{15}\right)$	${\dot{E}}_{D,cond}={\dot{E}}_{F,cond}-{\dot{E}}_{P,cond}$	$\varepsilon_{cond}=\frac{{\dot{E}}_{P,cond}}{{\dot{E}}_{F,cond}}$
System	-	$COP=\frac{{\dot{Q}}_{evap}}{{\dot{W}}_{LTC}+{\dot{W}}_{MTC}+{\dot{W}}_{HTC}}$	${\dot{E}}_{F,system}={\dot{E}}_{F,LTC,comp}+{\dot{E}}_{F,MTC,comp}+{\dot{E}}_{F,HTC,comp}$	${\dot{E}}_{P,sys}={\dot{E}}_{P,evap}$	${\dot{E}}_{D,sys}={\dot{E}}_{F,sys}-{\dot{E}}_{P,sys}-{\dot{E}}_{L,sys}$	$\varepsilon_{sys}=\frac{{\dot{E}}_{P,sys}}{{\dot{E}}_{F,sys}}$

Note that exergy loss means the transfer of exergy of the system (${\dot{E}}_{L,\;sys}$) as a whole to its surroundings [58]. The exergy loss is associated with the rejection of heat and material streams to the surroundings, which are not reused by the system, such as the condenser [56,58,60].

Table 5 lists the correlations for equipment costs used for all components in the TSCRS, as well as their updated coefficients, to reflect the current economic conditions as given by the current CEPCI. Table 6 lists all cost balance equations and auxiliary relations required to solve for all unknown cost rates in each stream in the system. These are a set of coupled algebraic equations whose simultaneous solution yields all unknown cost rates in each stream in the system. Consequently, not only is the unit exergy cost associated with each thermodynamic stream in the cycle determined but so is the total product cost rate of the system.

### 2.6. Intermediate Temperature Allocation in CHX Units and Optimization Strategy

The intermediate temperature, which refers to the condensation temperature inside the CHX of the LTC and MTC, has always been identified as an important parameter that influences the entire performance of TSCRSs [27] and CRSs [2]. This intermediate temperature largely influences the pressure ratio, efficiency, and work of the compressors used in the system [65]. This importance stems from the fact that the structural configuration of a TSCRS has the CHX of both the LTC and the MTC simultaneously performing the roles of condensation for the lower stage and evaporation for the upper stage. These thermodynamic interfaces between adjacent circuits are responsible for controlling the heat transfer interaction and matching of pressures and temperatures for the cascade levels. Therefore, the condensation temperature has a direct impact on the work of the compressor, the formation of irreversibility in the system, and thermodynamic efficiency, and it is an important optimization parameter for TSCRSs [27].

The LTC and MTC condenser temperatures are optimized using the DIRECT algorithm in EES. The direct search method is generally accepted as appropriate for simulation-based optimization and optimization of non-numerical functions [66]. The DIRECT algorithm is found to effectively locate conditions that result in the minimum total product cost rate due to its ability to perform a global search as a derivative-free method. Based on the given LTC and MTC temperature ranges, the optimum LTC and MTC condenser temperatures are determined for each pair of the LTC evaporator and HTC condenser temperatures by minimizing the total product cost rate of the TSCRS. This means that for all operating conditions of the lower and upper cycles, the optimum LTC and MTC condenser temperatures corresponding to the minimum total product cost rate are determined. For all specified temperatures of the LTC evaporator and HTC condenser, the optimal values of the LTC condenser temperature and MTC condenser temperature are determined based on the minimization of the total product cost rate, subject to the constraints on allowable temperature ranges and the requirement that all compressor discharge temperatures of the LTC, MTC and HTC remain below 120 °C:

$$\min{\dot{C}}_{P,sys}\left(T_{LTC,cond},T_{MTC,cond}\right)$$

subject to the following constraints:

$$-85<T_{LTC,cond}\left({}^{\circ}C\right)<-40$$

$$-40<T_{MTC,cond}\left({}^{\circ}C\right)<0$$

$$T_{i,comp}<120\;{}^{\circ}C\;(i=LTC,\;MTC\;or\;HTC)$$

## 3. Results and Discussion

In this section, the model validation is performed, in which the accuracy and reliability of the developed model are assessed by comparing the obtained results with the existing theoretical and experimental studies in the open literature. This ensures that the obtained results from the model are consistent with the previously validated studies of VCRSs, CRSs and TSCRSs. Subsequently, the optimal operating conditions of the TSCRS are obtained. Considering the decision variables introduced in Section 2.6, the optimal values of the condensation temperatures of the LTC and the MTC are obtained using the optimization procedure. This minimizes the product cost rate of the system, thereby assessing the exergoeconomic performance of the TSCRS. After the optimal operating conditions of the system are obtained, the exergy and exergoeconomic analyses of the TSCRS are carried out at fixed operating conditions. This step is useful in the evaluation of the exergy destructions associated with the TSCRS components, the mechanism of cost formation, and the relative significance of the investment and operation-maintenance cost and exergy destruction costs within the TSCRS. Lastly, a detailed parametric analysis is carried out to examine the effects of significant operating parameters on the exergy and exergoeconomic performance of the TSCRS. This results in greater insights into the system behavior and helps in the identification of the performance characteristics for varying operating conditions.

### 3.1. Model Validation

In view of the lack of available experimental data for TSCRSs, a full validation of the current model cannot be performed. Therefore, aside from comparisons among theories, the fundamental thermodynamic behavior of the current model was experimentally validated by utilizing experimental data from a well-established single-stage VCRS and a two-stage CRS. This indirect method of validation was likewise applied in previous studies, like that of Pektezel and Ozdemir (2026) [23], Nabil et al. (2023) [26] and Kayes et al. (2024) [54], where there were no experimental benchmarks for configurations greater than two stages.

In the absence of published experimental studies on TSCRSs, the validation of the developed model was initially carried out for a conventional VCRS, whose performance was experimentally investigated by Ma and Zhou (2008) [67]. In the present study, the operating conditions used by Ma and Zhou (2008) [67] were implemented, and the same configuration was simulated using the developed EES model. This helped in carrying out a preliminary evaluation of the model’s predictive ability before the analysis was carried out for the TSCRS configuration. Model validation is performed under the same operation conditions as with Ma and Zhao (2008) [67]: the refrigerant is R134a; T_cond_ is 40 °C; degree of subcooling in the condenser is 2.4 °C; degree of superheating in the evaporator is 10.6 °C; and compressor efficiencies are 63%, 54% and 45% at Tevap values of −15 °C, −10 °C and −5 °C, respectively. As can be seen in Table 7, using $\frac{\left|{Parameter}_{present}-{Parameter}_{ref}\right|}{{Parameter}_{ref}}$, the maximum relative deviation between the present results and the reference data is 0.93%, which confirms a high level of agreement and ensures the accuracy of the proposed model.

The validation of this study is supported by the results obtained by Dopazo and Fernández-Seara (2011) [68] using $\frac{\left|{COP}_{present}-{COP}_{ref}\right|}{{COP}_{ref}}$, as shown in Table 8. The two-stage CRS was operated at an evaporator temperature of −50 °C, whereas the LTC system’s CO_2_ condenser temperature ranged from −17 °C to −10 °C. Additionally, the superheating of the CO_2_ system was set at 15 °C, with subcooling at 0 °C. The HTC system’s NH_3_ side condenser temperature was set at 30 °C, with superheating at 15 °C and subcooling at 5 °C. Based on the system’s operation under these conditions, the COP values calculated in this study were found to correspond to the experiment’s measurements with a maximum deviation of 3.6%.

The validation of this study is supported by the results obtained by Nabil et al. (2023) [26] using $\frac{\left|{Parameter}_{present}-{Parameter}_{ref}\right|}{{Parameter}_{ref}}$, as shown in Table 9. The thermodynamic results for the TSCRS model presented by Nabil et al. (2023) [26] were obtained under the same operating conditions and compared with the results obtained from the present study. The performance parameters, such as condenser load, compressor work, COP, and exergy efficiency, were considered for the validation of the results. Model validation is performed under the same operation conditions as with Nabil et al. (2023) [26]: refrigerant: m-Xylene (LTC), toluene (MTC), and 1-butene (HTC), dead state pressure of 101.325 kPa, dead state temperature of 25 °C, evaporator temperature of −100 °C, condenser temperature of 40 °C, and temperature difference in the CHX of 5 °C and refrigeration capacity of 10 kW. The percentage deviation varies from 0.17% to 0.53%. The close agreement between the results obtained from the present study and the results presented by Nabil et al. (2023) [26] confirms the validity of the developed model. Based on the operation conditions reported in Nabil et al. (2023) [26], the current system was simulated using the proposed EES-based thermodynamic model.

As presented in Table 10, using $\frac{\left|{COP}_{present}-{COP}_{ref}\right|}{{COP}_{ref}}$, there is an excellent concordance between the present TSCRS model and the theoretical results provided by Sun et al. (2019) [27]. In fact, the maximum relative deviation is 0.16%, and the trend of the COP versus the LTC condenser temperature is correctly reproduced. These results confirm the consistency and numerical stability of the proposed EES model. In terms of operating conditions, refrigerants are chosen to be R1150/R170/R161, refrigeration capacity is 10 kW, temperature difference in CHX is 5 °C, T_evap_ = −100 °C, T_cond_ = 40 °C, HTC superheating is 5 °C, and superheating of both LTC and MTC is 5 °C. In this context, the performance of the R1150/R170/R161 configuration is evaluated under a trustworthy modeling framework.

Table 11 shows the validation of the TSCRS model’s total cost rate against the theoretical results provided by Kayes et al. (2024) [54] using $\frac{\left|{Total\;cost\;rate}_{present}-{Total\;cost\;rate}_{reference}\right|}{{Total\;cost\;rate}_{reference}}$. In terms of operating conditions, the refrigerants chosen are 1-butene/heptane/m-xylene, the refrigeration capacity is 10 kW, the temperature difference in CHX is 5 °C, T_evap_ = −120 °C, T_cond_ = 40 °C, and T_MTC_ = −30 °C. As demonstrated, there is a high degree of similarity over the entire range of the LTC condenser temperature. The percentage deviation varies from 0.25% to 0.92%. Apart from the minor numerical differences, it is worth mentioning that the proposed model is able to produce the same cost trend as a function of the LTC condenser temperature, thus verifying the correct application of the exergoeconomic model and cost balance equations. The minor underestimation of the proposed model does not affect the results in any way and is within an acceptable engineering accuracy. To conclude, the high degree of similarity between this study and Kayas et al. (2024) [54] verifies the accuracy and robustness of the proposed model in terms of predicting the total cost rate of the TSCRS.

The comparison is carried out with the findings of the present research work and those of Johnson et al. (2017) [46] at the same operating conditions, with the evaporator temperature being −163 °C and the condenser temperature being 50 °C. In addition to this, the cascade temperature difference is kept at 5 °C, while the polytropic efficiency of the compressor is taken as 0.8. From Table 12, using $\frac{\left|{COP}_{present}-{COP}_{ref}\right|}{{COP}_{ref}}$, the relative percentage deviations in the COP_LT_, COP_MT_, COP_HT_, and COP values are found to be 0.0031%, 0.23%, 1.078%, and 0.61%, respectively. All these values are extremely low and are well within the range of below 1.1%. Therefore, the findings confirm the numerical accuracy, thermodynamic correctness, and reliability of the proposed simulation model for the TSCRS.

### 3.2. Results of Exergy and Exergoeconomic Analysis

The importance of this study can be gauged from the fact that it provides a thorough analysis of the impact of the choice of refrigerants on exergy destruction, cost formation, and performance of TSCRSs in terms of exergy and exergoeconomics.

#### 3.2.1. Determination of Optimal LTC and MTC Condenser Temperatures

**Figure 2:**
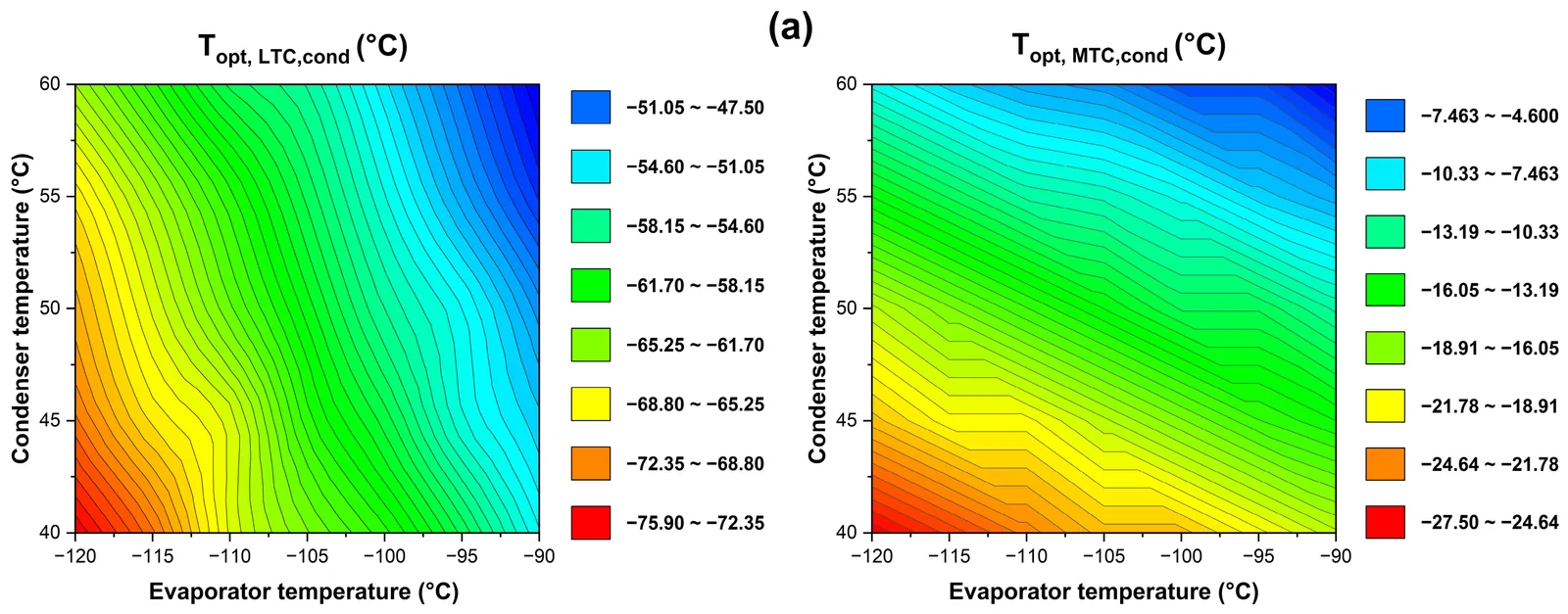

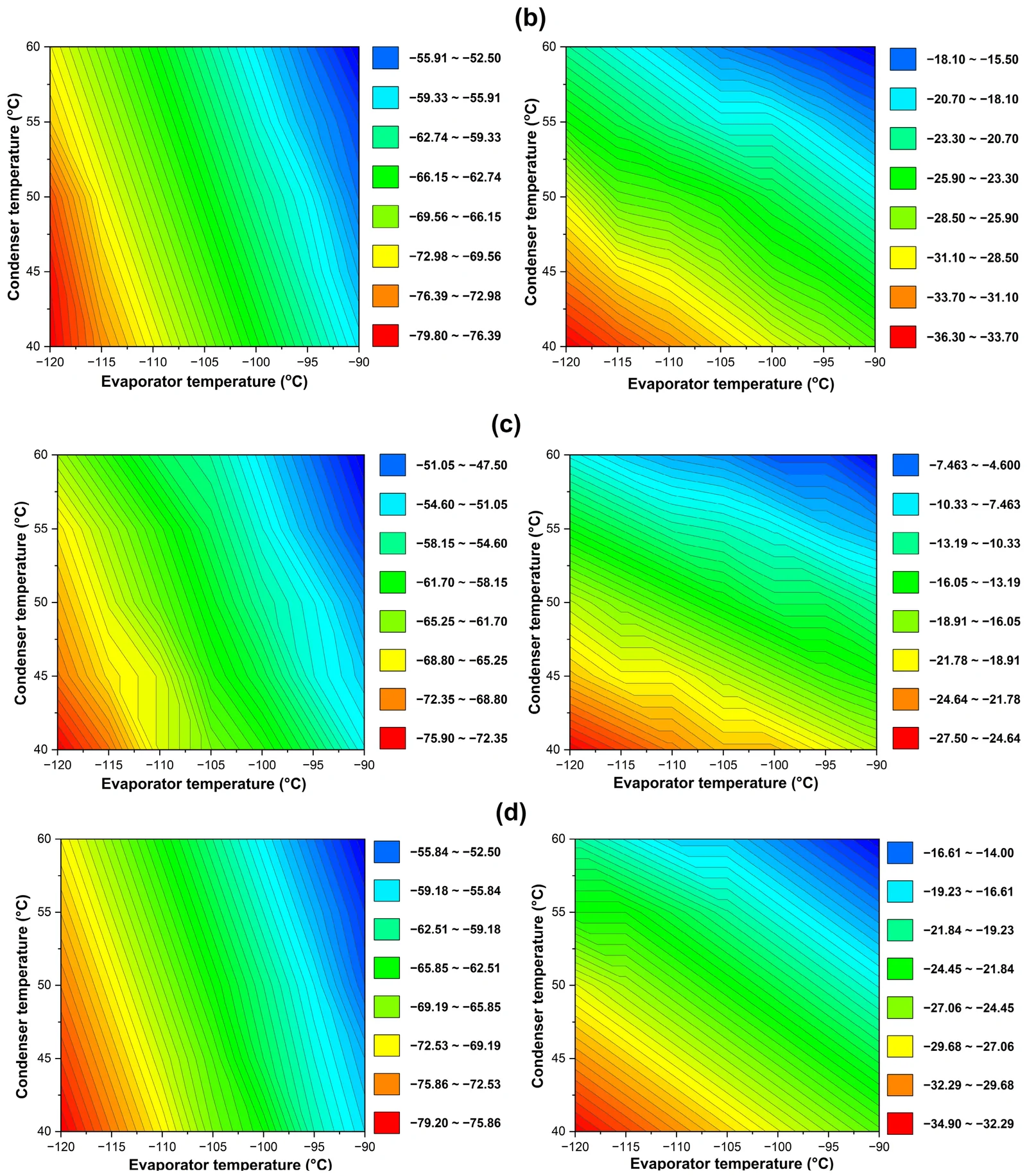
Contour plots illustrating the sensitivity of the TSCRS to evaporator and condenser temperatures: (**a**) R1150/R170/R1270, (**b**) R1150/R170/R152a, (**c**) R1150/R170/R290, (**d**) R1150/R170/R161.

Figure 2 illustrates the optimal LTC and MTC condenser temperatures as functions of the evaporator temperatures ranging from −120 to −90 °C and condenser temperatures ranging from 40 to 60 °C for R1150/R170/R1270, R1150/R170/R152a, R1150/R170/R290 and R1150/R170/R161. It can be seen that, in all cases, the optimal temperatures of the LTC and MTC both increase as the evaporator and condenser temperatures increase. R1150/R170/R1270 has the optimal LTC condenser temperature ranging from −75.83 to −49.17 °C, whereas the optimal MTC condenser temperature extends from −28.89 to −6.67 °C. If R152a is used in the HTC, the optimal temperatures of the LTC and MTC both decline, where the optimal LTC condenser temperature varies from −79.72 to −52.50 °C, and the optimal MTC condenser temperature falls within the range of −36.30 to −15.56 °C. A similar trend can also be found in the R1150/R170/R161 refrigerant combination, where the optimal LTC condenser temperature ranges from −79.17 to −52.50 °C, and the optimal MTC condenser temperature spans −34.81 to −14.07 °C. However, the R1150/R170/R290 combination has higher optimal temperatures, where the LTC condenser temperature ranges from −75.83 to −47.50 °C, and the MTC condenser temperature varies from −27.41 to −4.69 °C. It can be concluded that increasing the evaporator and condenser temperatures has resulted in increasing the optimal cascade stage temperatures. Since the refrigerant used in the LTC and MTC stages has been fixed, it can be noted that the difference in temperatures has resulted from the refrigerant used in the HTC. It can be noted that the thermophysical properties of the refrigerant used in the HTC have affected the temperature distribution in the TSCRS.

#### 3.2.2. Component-Wise Analysis

Table 13, Table 14, Table 15 and Table 16 present a summary of the thermodynamic and exergoeconomic characteristics of the TSCRS over all state points for the different combinations of refrigerants. In the LTC, the evaporator operates at −100 °C (state 1) with an inlet pressure to the compressor of approximately 126 kPa. The refrigerant rejected from the LTC compressor gets condensed in the CHX at approximately −60 to −64 °C, which corresponds to state 3. This temperature also represents the evaporating temperature of the MTC. The MTC condenser temperature is approximately −18 to −27 °C, which corresponds to state 7. This temperature also represents the evaporating temperature of the HTC. The HTC rejects heat to the environment in the condenser at approximately 45 °C, which corresponds to state 11. The mass flow rate of the LTC remains almost constant for all refrigerant combinations, spanning 0.129 to 0.133 kg/s. The MTC has a mass flow rate ranging from 0.189 to 0.209 kg/s. The HTC has the highest mass flow rate, varying from 0.348 to 0.430 kg/s depending on the refrigerant used in the cycle. The exergy flow rate also increases in the compression process and reaches its maximum in the HTC compressor. The cost analysis shows that the unit exergy cost of the refrigerant changes as it passes through the compressors and CHXs that interconnect the stages of the system. The air streams in the condenser and evaporator sections are assigned a unit exergy cost of zero since they are classified as environmental streams. The thermodynamic structure of the cycle remains the same for all the refrigerant combinations, and variations in the mass flow rate, exergy flow rate, and unit cost rate are due to the thermophysical properties of the refrigerant used in the HTC.

**Figure 3:**
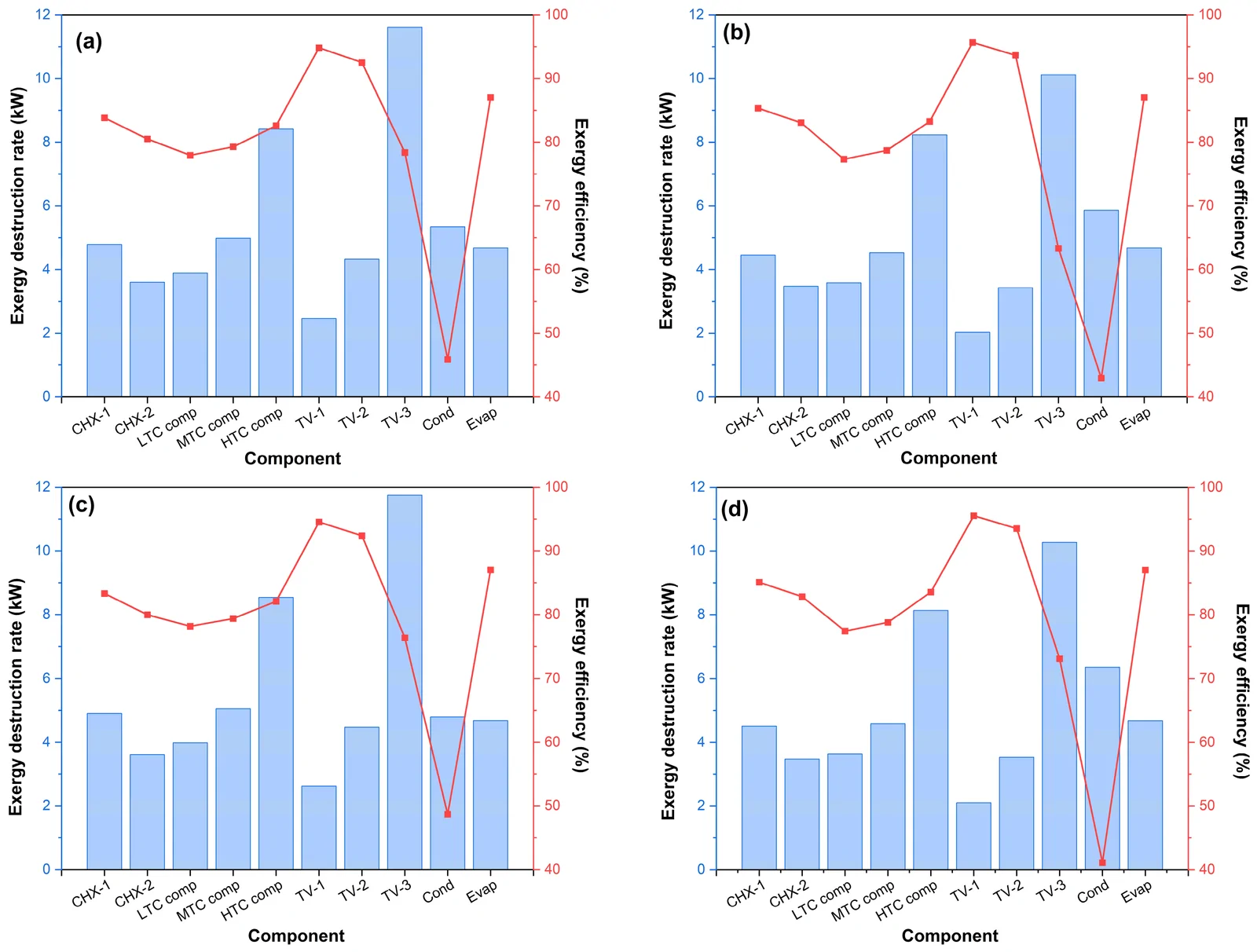
Exergy destruction rate and exergy efficiency of each component of TSCRS: (**a**) R1150/R170/R1270, (**b**) R1150/R170/R152a, (**c**) R1150/R170/R290, (**d**) R1150/R170/R161.

Figure 3 presents a diagram of component exergy destruction and component exergy efficiency for the TSCRS for the different refrigerant combinations at an evaporator temperature of −100 °C and a condenser temperature of 45 °C. The results reveal that the highest exergy destruction occurs in TV-3 and the HTC compressor. The exergy destruction rate of TV-3 is 11.61 kW, 10.11 kW, 11.75 kW, and 10.27 kW for R1150/R170/R1270, R1150/R170/R152a, R1150/R170/R290, and R1150/R170/R16, respectively. The HTC compressor also has considerable exergy destruction, and its values are 8.415 kW, 8.23 kW, 8.54 kW, and 8.132 kW. The exergy destruction values for the condenser are 5.343 kW, 5.86 kW, 4.794 kW, and 6.358 kW. The exergy destruction values for the CHXs are moderate, and the values for CHX-1 and CHX-2 are between 4.451 kW and 4.902 kW and between 3.471 kW and 3.607 kW, respectively. The exergy destruction values for LTC and MTC compressors are between 3.58 kW and 3.981 kW and between 4.525 kW and 5.051 kW, respectively. The exergy destruction value of the evaporator is constant at 4.68 kW for all refrigerant combinations since the operating conditions of the LTC remain unchanged. The exergy efficiency results show a consistent relationship with the distribution of exergy destruction rate. The range of exergy efficiencies in the CHXs varies from 83.31% to 85.34% in CHX-1 and from 79.98% to 83.08% in CHX-2. The span of exergy efficiencies in the compressors varies from 77.32% to 77.96% in the LTC, from 78.72% to 79.37% in the MTC, and from 82.08% to 83.55% in the HTC. The range of exergy efficiencies in TV-1 and TV-2 is relatively high, and varies from 94.53% to 95.68% in TV-1 and from 92.39% to 93.66% in TV-2. The range of efficiencies in TV-3 is relatively low, from 63.32% to 78.39%. The lowest exergy efficiency is observed in the condenser, varying from 41.11% to 48.66%. The refrigerants in the LTC and MTC are constant, and the changes in the refrigerant in the HTC are responsible for the observed differences in the exergy destruction in the components of the TSCRS.

**Figure 4:**
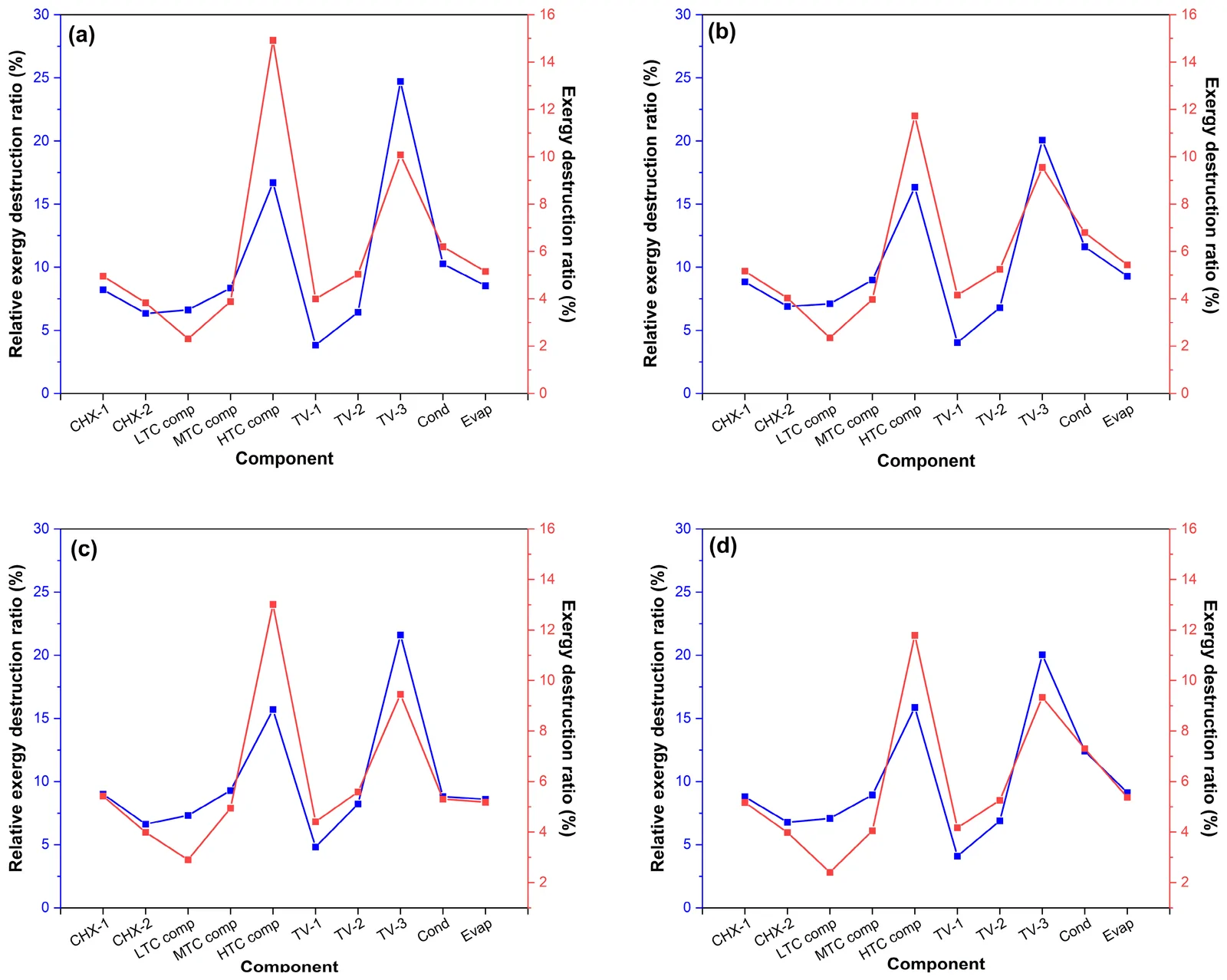
Relative exergy destruction ratio and exergy destruction ratio of each component of TSCRS: (**a**) R1150/R170/R1270, (**b**) R1150/R170/R152a, (**c**) R1150/R170/R290, (**d**) R1150/R170/R161.

Figure 4 shows a plot of the relative exergy destruction ratio and exergy destruction ratio for each component in the TSCRS for different refrigerant combinations under an evaporator temperature of −100 °C and a condenser temperature of 45 °C. It can be seen that for all refrigerant combinations, the highest amount of exergy destruction is associated with TV-3 and the HTC compressor. For R1150/R170/R1270, the relative exergy destruction ratio for TV-3 is found to reach a value of 24.71%, which is the highest for all components in the system. The second-highest exergy destruction is associated with the HTC compressor, which is found to contribute 16.70% to the total exergy destruction. Similarly, for other refrigerant combinations, the highest amount of exergy destruction is associated with TV-3 and the HTC compressor. For R1150/R170/R152a, TV-3 is found to contribute 20.08% to the total exergy destruction, while the HTC compressor contributes 16.34%. The corresponding values for R1150/R170/R290 are 21.61% and 15.70%, while for R1150/R170/R161, these values are 20.04% and 15.87%, respectively. The condenser also contributes to the total exergy destruction rate, where the relative exergy destruction ratios of 10.27%, 11.63%, 8.812%, and 12.41% correspond to the four different combinations of refrigerants. The evaporator has a relative exergy destruction ratio in the range of 8.535% to 9.292%, which indicates that the level of exergy destruction is moderate in the heat absorption process. Regarding the CHXs, the relative exergy destruction ratios range from 6.344% to 9.011% for CHX-1, whereas the relative exergy destruction ratios of CHX-2 range from 6.344% to 6.896%. The relative exergy destruction ratios of the LTC and MTC compressors are comparatively low, ranging from 6.621% to 7.318% and 8.352% to 9.285%, respectively. The relative exergy destruction ratios of TV-1 are the lowest among all the components, ranging from 3.827% to 4.815%. From the exergy destruction ratios, it is observed that the HTC compressor is found to be a major contributor to exergy destruction compared to the total fuel exergy supplied to the system. The exergy destruction ratio for this compressor is found to be 14.92%, 11.73%, 13.01%, and 11.79% for R1150/R170/R1270, R1150/R170/R152a, R1150/R170/R290, and R1150/R170/R161, respectively. TV-3 also has a significant contribution to total exergy destruction and is found to be 10.08%, 9.549%, 9.452%, and 9.336% for the above refrigerants, respectively. The contribution of the condenser ranges from 5.305% to 7.299%, while that of the evaporator remains constant and ranges between 5.154% and 5.43%. This is because the operating conditions of the LTC remain unchanged. The exergy destruction ratios for CHX-1 range from 4.962% to 5.425%, and those for CHX-2 range from 3.831% to 4.03%. The compressors of the LTC and MTC have smaller contributions and range between 2.311% and 2.899% and between 3.885% and 4.947%, respectively. The exergy destruction ratios for TV-1 and TV-2 are found to be 3.998% to 4.406% and 5.043% to 5.59%, respectively. It can be concluded that the compression process in the HTC and the expansion process in TV-3 are major contributors to exergy destruction compared to the total fuel exergy supplied to the TSCRS.

**Figure 5:**
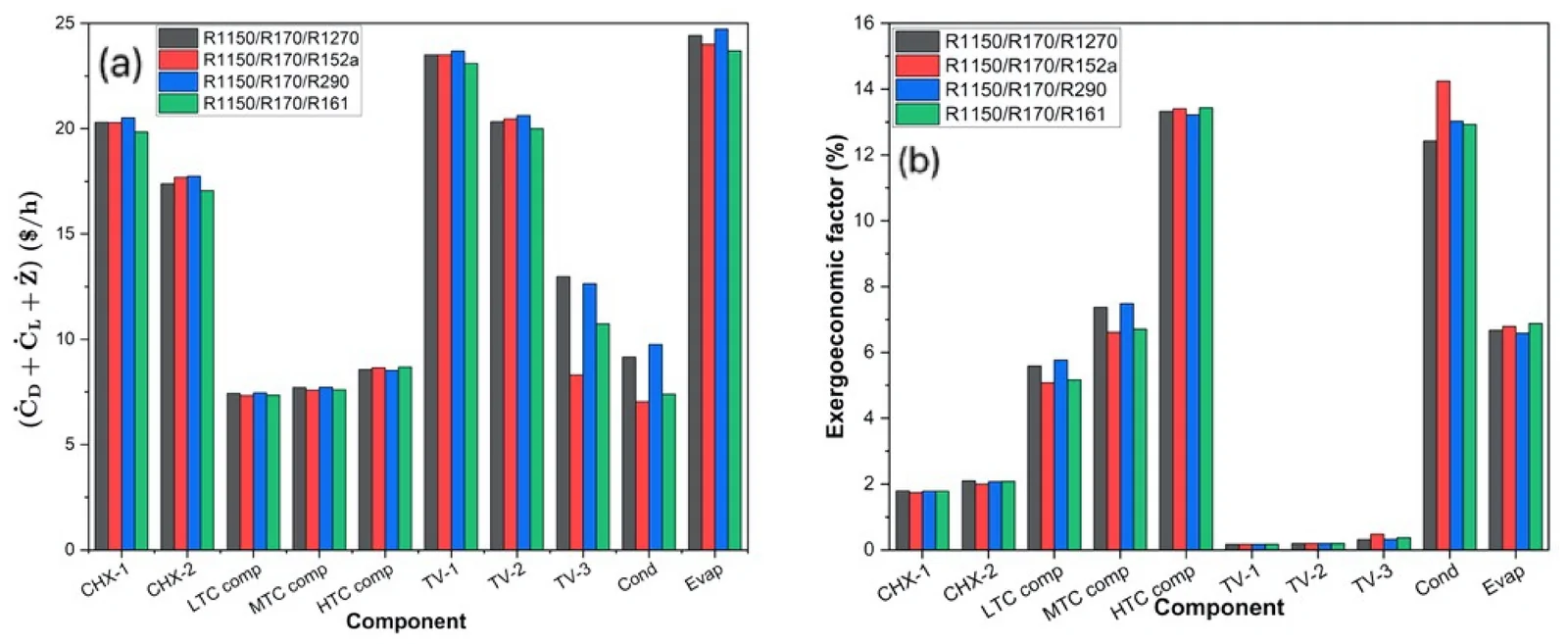
Exergoeconomic assessment of the TSCRS components: (**a**) cost distribution; (**b**) exergoeconomic factor (T_evap_ = −100 °C and T_cond_ = 45 °C).

Figure 5 shows the exergoeconomic evaluation of the components in the TSCRS, in terms of total cost rate and exergoeconomic factor, for the four different refrigerant pairs at an evaporator temperature of −100 °C and a condenser temperature of 45 °C. Table 17 lists the individual cost rate contributions for each component, in terms of ${\dot{Z}}_{k}$, ${\dot{C}}_{D,k}$, and ${\dot{C}}_{L,k}$. The totals of ${\dot{Z}}_{k}$, ${\dot{C}}_{D,k}$, and ${\dot{C}}_{L,k}$ in Figure 5a are the summation of all the individual cost rate contributions for the components. The cost rate distribution implies that the evaporator and TV-1 have the highest cost rates in the system. The evaporator ranges from 23.69 $/h to 24.73 $/h, depending on the combination of the refrigerant, whereas TV-1 ranges from 23.10 $/h to 23.68 $/h. The CHXs also contribute significantly to the cost rate in the system. CHX-1 ranges from 19.85 $/h to 20.52 $/h, whereas CHX-2 ranges from 17.06 $/h to 17.74 $/h. The compressor cost rates increase progressively along the cascade stages, ranging from 7.33 to 7.46 $/h for the LTC compressor, 7.59 to 7.72 $/h for the MTC compressor, and 8.51 to 8.69 $/h for the HTC compressor. The condenser and TV-3 have moderate cost rate values in the system, ranging from 7.03 $/h to 9.75 $/h and 8.31 $/h to 12.98 $/h, respectively. The exergoeconomic factor in Figure 5b provides further information on the cost rate distribution of each component. The HTC compressor has the highest exergoeconomic factor, ranging from 13.22% to 13.44%, followed by the condenser, which varies from 12.42% to 14.24%. The evaporator varies from 6.59% to 6.88%, and the LTC and MTC compressors vary from 5.09% to 5.77% and from 6.62% to 7.49%, respectively. On the other hand, the exergoeconomic factors of the expansion valves (TV-1, TV-2 and TV-3) are found to be small. TV-1 varies from 0.170% to 0.175%, TV-2 varies from 0.196% to 0.202%, and TV-3 varies from 0.311% to 0.485%.

**Figure 6:**
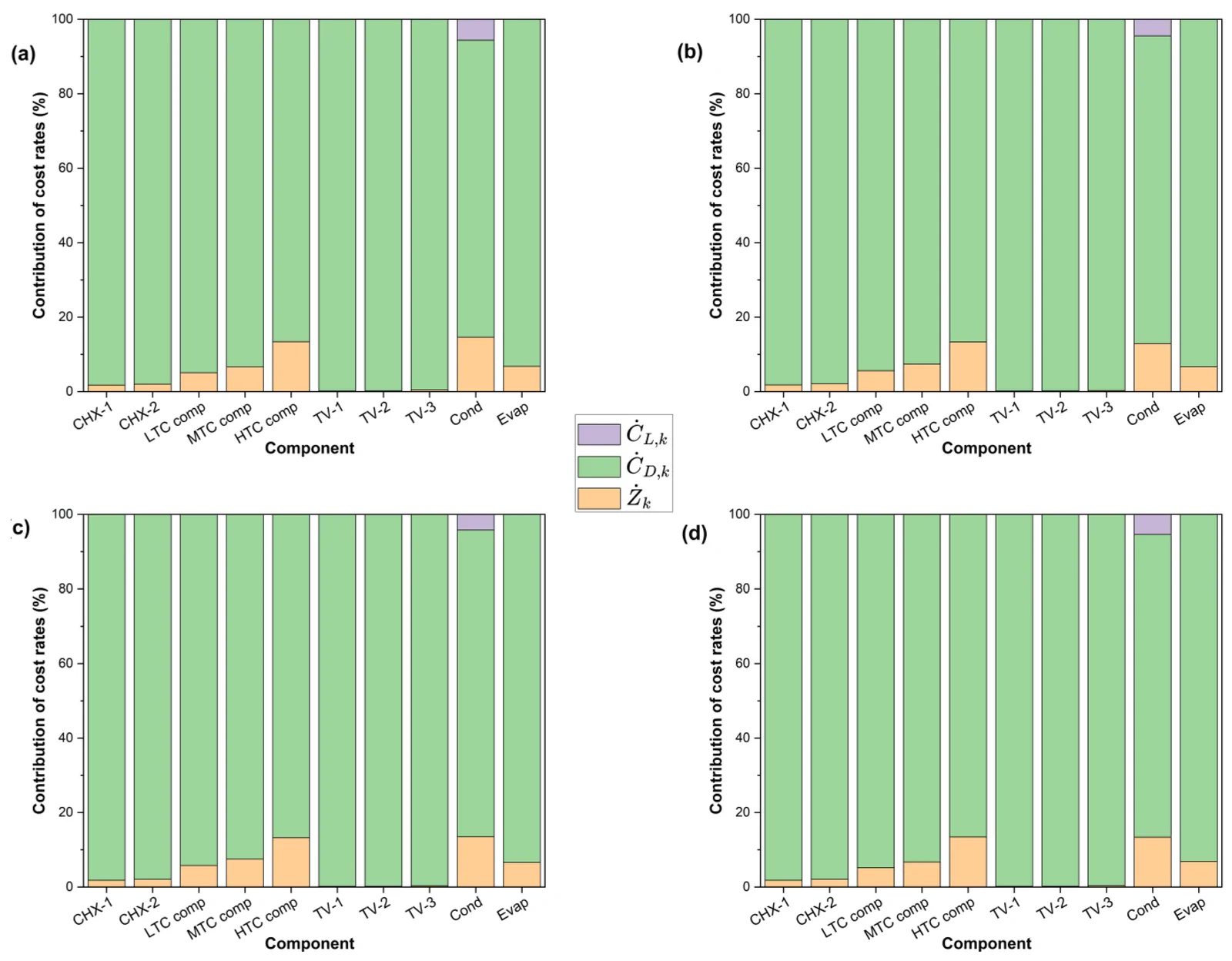
Cost dominance distribution of Ż_k_, Ċ_D,k_, and Ċ_L,k_ for the components of the TSCRS under different refrigerant combinations: (**a**) R1150/R170/R1270, (**b**) R1150/R170/R152a, (**c**) R1150/R170/R290, and (**d**) R1150/R170/R161.

The cost dominance distribution of the system components is shown in Figure 6. The results show that the cost structure of all components is largely influenced by the exergy destruction cost rate for all the refrigerant combinations. For CHX-1, Ċ_D,k_ is found to vary between 98.20 and 98.25%. Ż_k_ is still at a minimum, ranging from 1.75 to 1.80%, while Ċ_L,k_ is 0%. A similar trend is observed for CHX-2, where Ċ_D,k_ varies from 97.90 to 98.00%. Ż_k_ is still at a minimum, ranging from 2.00 to 2.10%. This shows that the total cost rate of the two heat exchangers (CHX-1 and CHX-2) is largely controlled by Ċ_D,k_. In the compressors, the contribution of Ż_k_ is more significant, though Ċ_D,k_ still influences the total cost rate. In the LTC compressor, the contribution of Ż_k_ is found to vary from 5.09 to 5.77%. Ċ_D,k_, on the other hand, varies from 94.23 to 94.91%. In the MTC compressor, the contribution of Ż_k_ is slightly higher, ranging from 6.62 to 7.49%. Ċ_D,k_ is still the major contributor, ranging from 92.51 to 93.38%. In the HTC compressor, the contribution of Ż_k_ is the highest, ranging from 13.22 to 13.43%. Ċ_D,k_ is still the major contributor, ranging from 86.57 to 86.78%. For the expansion valves (TV-1, TV-2, and TV-3), the contribution of Żk is exceedingly low. In TV-1, Ż_k_ is 0.17%, while Ċ_D,k_ is 99.83% of the total cost rate for all combinations of refrigerants. In TV-2, Ż_k_ ranges between 0.19% and 0.20%, with Ċ_D,k_ ranging between 99.80% and 99.81%. In TV-3, Ż_k_ ranges between 0.31% and 0.48%, with Ċ_D,k_ ranging between 99.52% and 99.69%. This clearly indicates that the total cost rate of the expansion valves is almost entirely dominated by Ċ_D,k_. In the condenser component, Ż_k_ ranges between 12.88% and 14.61%, Ċ_D,k_ ranges between 79.75% and 82.66%, while Ċ_L,k_ ranges between 4.19% and 5.64%. Finally, with respect to the cost contribution for the evaporator component, Ż_k_ ranges between 6.59% and 6.88%, Ċ_D,k_ ranges between 93.12% and 93.41%, and Ċ_L,k_ is 0% for all combinations of refrigerants. The percentage contributions remain nearly consistent across all four refrigerant combinations. This indicates that the cost dominance pattern of the system components is only weakly affected by the refrigerant combinations used. Ċ_D,k_ is the dominant cost rate term in almost all system components, while Ż_k_ becomes more significant in the evaporator, compressor and condenser components, and Ċ_L,k_ is significant only in the condenser component. Also, the cost dominance classification for each component is shown in Table 18.

#### 3.2.3. Parametric Analysis

**Figure 7:**
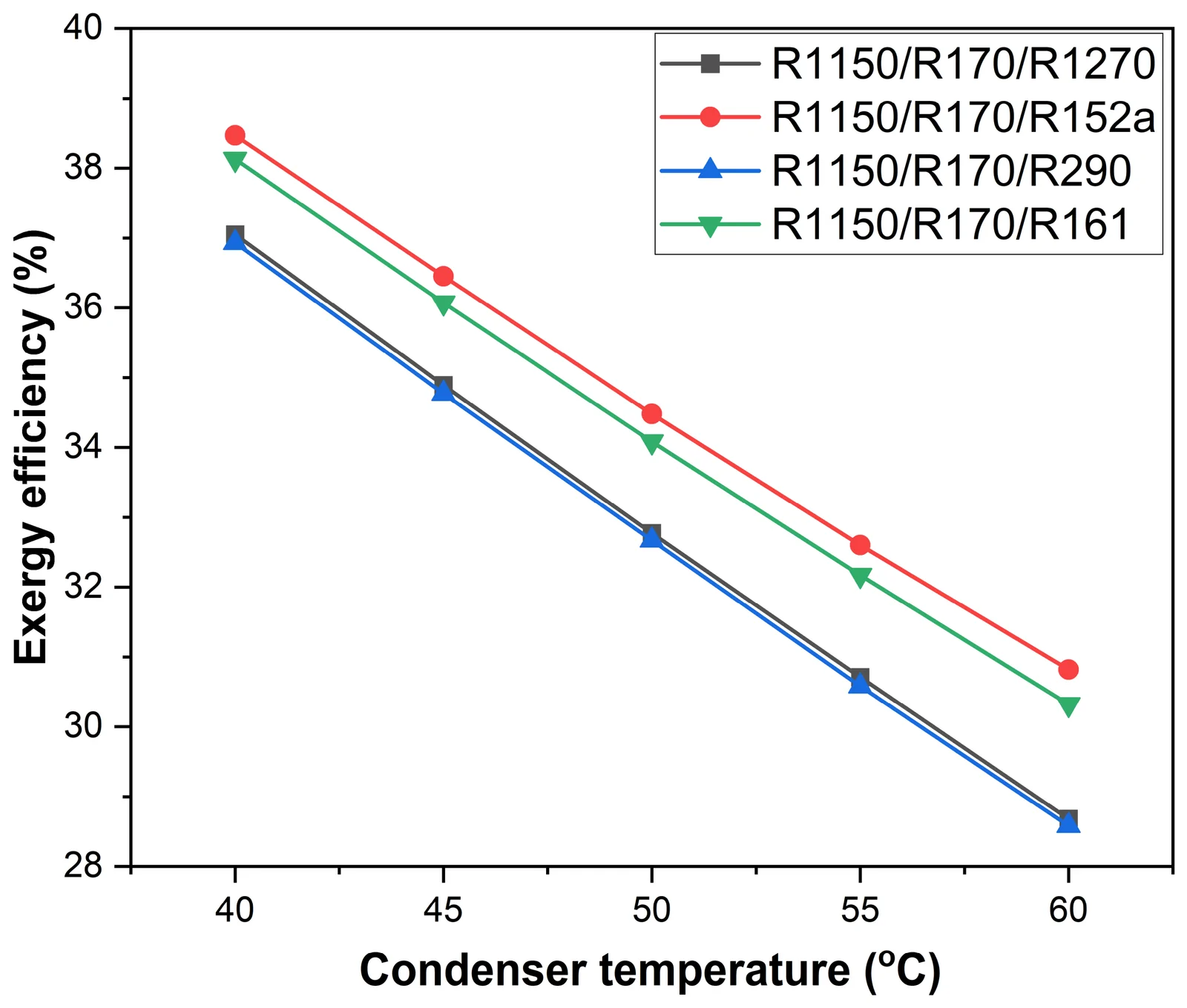
Alteration of the exergy efficiency illustrating the sensitivity of the TSCRS to condenser temperature.

The variation in system exergy efficiency with the condenser temperature for various refrigerant combinations is presented in Figure 7 at a fixed evaporator temperature of −100 °C. For all combinations, the exergy efficiencies decrease monotonically with an increasing condenser temperature, from 40 °C to 60 °C. The increase in condenser temperature leads to an increase in the condensate pressure of the HTC cycle, which increases the pressure ratio and power required by the compressor. Consequently, thermodynamic irreversibilities increase, particularly in compressors and cascade heat exchangers, resulting in greater total exergy destruction. Since a larger portion of the supplied exergy is destroyed within the system, overall exergy efficiency decreases with increasing condenser temperature. Among all the refrigerant combinations, the exergy efficiency of R1150/R170/R152a is the highest at all condenser temperature ranges, ranging from 38.47% at a temperature of 40 °C down to 30.82% at a temperature of 60 °C. A similar trend is also seen in the case of R1150/R170/R161, where exergy efficiency varies from 38.13% at a condenser temperature of 40 °C down to 30.32% at a condenser temperature of 60 °C. On the other hand, the exergy efficiency of the combinations involving R1150/R170/R1270 and R1150/R170/R290 is lower at all temperature ranges. In the case of the combination involving R1150/R170/R1270, exergy efficiency varies from 37.05% at a condenser temperature of 40 °C down to 28.68% at a condenser temperature of 60 °C, while in the case of the combination involving R1150/R170/R290, exergy efficiency varies from 36.93% at a temperature of 40 °C down to 28.58% at a temperature of 60 °C. This shows that the combination involving R1150/R170/R152a has the maximum exergy efficiency, while the combination involving R1150/R170/R290 has the least exergy efficiency. It is also noteworthy that the ranking of the refrigerant combinations is the same throughout the whole range of the condenser temperature; i.e., R1150/R170/R152a > R1150/R170/R161 > R1150/R170/R1270 > R1150/R170/R290. This shows that the thermodynamic superiority of the HTC based on R152a and R161 is preserved even at more severe condensation conditions. The results show that the lower condenser temperatures improve the exergy efficiency, and the appropriate refrigerant used in the HTC is the key to improving the exergetic performance of the TSCRS.

**Figure 8:**
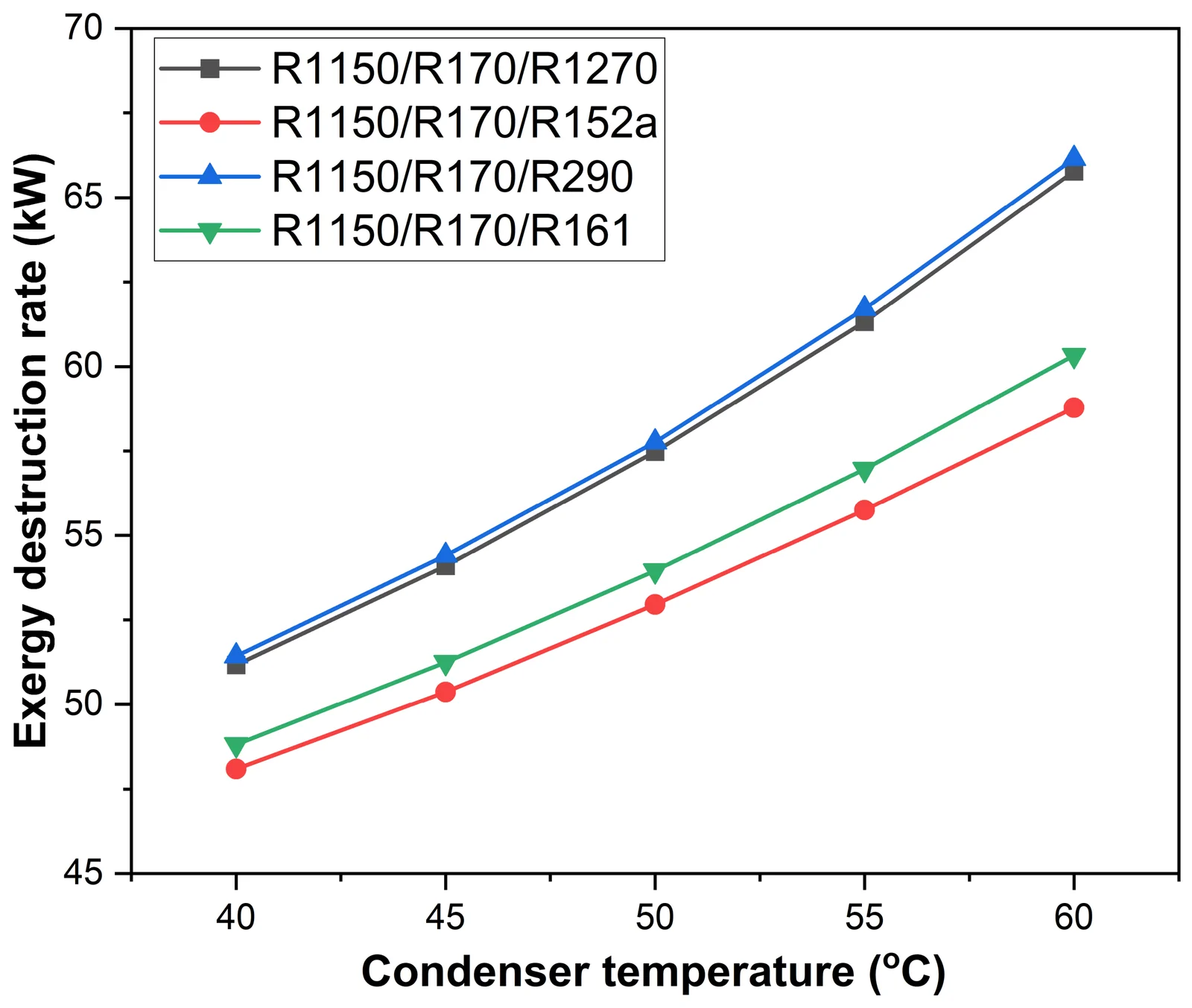
Alteration of the exergy destruction rate illustrating the sensitivity of the TSCRS to condenser temperature.

The variation in the total exergy destruction rate with condenser temperature for all the investigated refrigerant combinations is presented in Figure 8 at a fixed evaporator temperature of −100 °C. An increase in the condenser temperature from 40 °C to 60 °C results in a corresponding increase in the total exergy destruction rate for all the investigated refrigerant combinations, indicating the progressive increase in the destruction rate of the system. The increase in total exergy loss with increased condenser temperatures is primarily due to the presence of high irreversibilities in the compressors and heat exchangers. Higher temperatures in the condenser lead to higher amounts of work needed for compression, leading to higher temperature differences while transferring heat, hence more entropy generation and total exergy destruction. It is also observed that R1150/R170/R290 results in the highest exergy destruction rates for all the investigated temperature ranges, increasing from 51.43 kW at a temperature of 40 °C to 66.15 kW at a temperature of 60 °C. A similar trend is observed for R1150/R170/R1270, where the exergy destruction rate increases from 51.17 kW to 65.78 kW with an increase in the condenser temperature from 40 °C to 60 °C. On the other hand, R1150/R170/R152a results in the lowest exergy destruction rates for all the investigated temperature ranges, increasing from 48.09 kW to 58.78 kW with an increase in the temperature level of the condenser from 40 °C to 60 °C. R1150/R170/R161 results in intermediate values, increasing from 48.81 kW to 60.33 kW with an increase in the temperature level of the condenser from 40 °C to 60 °C. It is also observed that the relative ranking of all the investigated refrigerant combinations is similar for all the investigated temperature ranges, with R1150/R170/R290 > R1150/R170/R1270 > R1150/R170/R161150/R170/R152a, indicating the strong influence of the refrigerant combination used in the HTC on the exergy destruction of the system.

**Figure 9:**
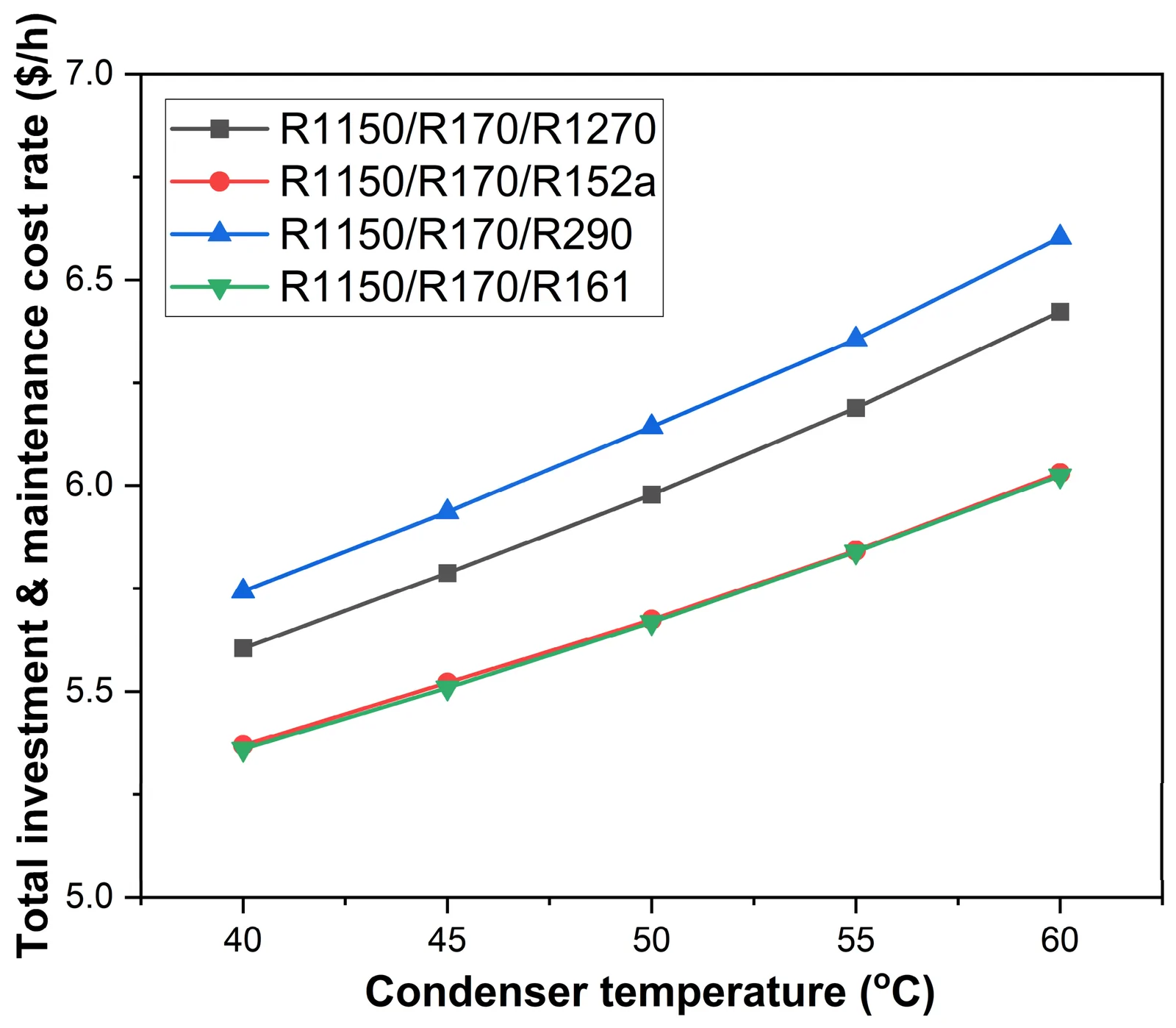
Alteration of the total investment & maintenance cost rate illustrating the sensitivity of the TSCRS to condenser temperature.

The variation in ${\dot{Z}}_{sys}^{CI\&OM}$ with the condenser temperature for the different refrigerant combinations is presented in Figure 9 at a fixed evaporator temperature of −100 °C. It is observed that with the increase in the condenser temperature from 40 °C to 60 °C, the ${\dot{Z}}_{sys}^{CI\&OM}$ increases monotonically for all the refrigerant configurations. As the temperature of the condenser increases, the power demand of the compressor and the heat transfer capacity required in the heat exchangers also rise. This requires a greater heat transfer area and a greater compressor capacity, leading to an increased ${\dot{Z}}_{sys}^{CI\&OM}$. Among the refrigerant combinations, R1150/R170/R290 has the highest ${\dot{Z}}_{sys}^{CI\&OM}$ over the entire range of temperature, increasing from 5.743 $/h to 6.603 $/h with the rise in temperature. A similar trend is also observed in R1150/R170/R1270, with the ${\dot{Z}}_{sys}^{CI\&OM}$ increasing from 5.605 $/h to 6.423 $/h with the rise in temperature. However, R1150/R170/R152a and R1150/R170/R161 have a relatively low ${\dot{Z}}_{sys}^{CI\&OM}$ under the given conditions. For R1150/R170/R152a, the ${\dot{Z}}_{sys}^{CI\&OM}$ increases from 5.369 $/h to 6.030 $/h, whereas the ${\dot{Z}}_{sys}^{CI\&OM}$ increases from 5.360 $/h to 6.024 $/h in R1150/R170/R161 configuration, with a similar trend. Even though the ${\dot{Z}}_{sys}^{CI\&OM}$ increases in all the refrigerant configurations with the rise in the condenser temperature, the R1150/R170/R161 and R1150/R170/R152a configurations have the minimum ${\dot{Z}}_{sys}^{CI\&OM}$, whereas the R1150/R170/R290 configuration has the highest ${\dot{Z}}_{sys}^{CI\&OM}$ in the entire range of temperature.

**Figure 10:**
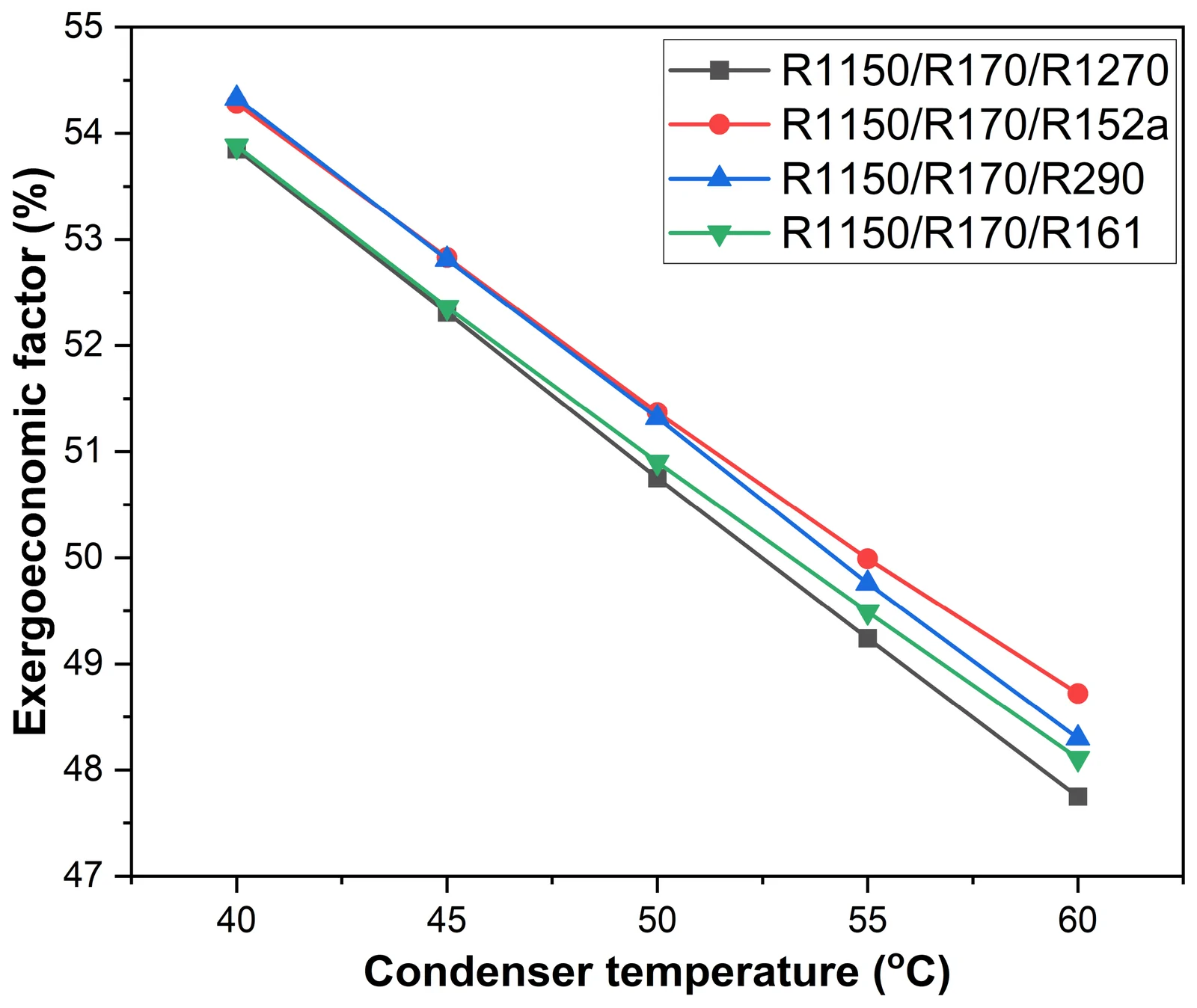
Alteration of the exergoeconomic factor illustrating the sensitivity of the TSCRS to condenser temperature.

The variation in the exergoeconomic factor with the condenser temperature for the investigated refrigerant combinations is presented in Figure 10 at a fixed evaporator temperature of −100 °C. Based on the definition mentioned in Equation (25), the observed decline in the exergoeconomic factor indicates a reduced relative contribution of capital investment and operation–maintenance costs, with the costs associated with the exergy destruction becoming more dominant. Among the investigated refrigerant combinations, R1150/R170/R290 presents the highest exergoeconomic factor values at lower condenser temperatures, reaching 54.33% at 40 °C, followed by R1150/R170/R152a, with an exergoeconomic factor value of 54.28%. R1150/R170/R1270 and R1150/R170/R161 present almost the same exergoeconomic factor values at the same temperature, ranging from 53.85 to 53.88%. At higher condenser temperatures, the exergoeconomic factor values decrease gradually for the investigated refrigerant combinations. At a condenser temperature pf 60 °C, the exergoeconomic factor values are 47.75%, 48.72%, 48.30%, and 48.11% for R1150/R170/R1270, R1150/R170/R152a, R1150/R170/R290, and R1150/R170/R161, respectively. The best cost structure is found for the combination based on R152a, while the combination based on R290 shows a greater predominance of exergy destruction-related costs, especially at high temperatures in the condenser.

**Figure 11:**
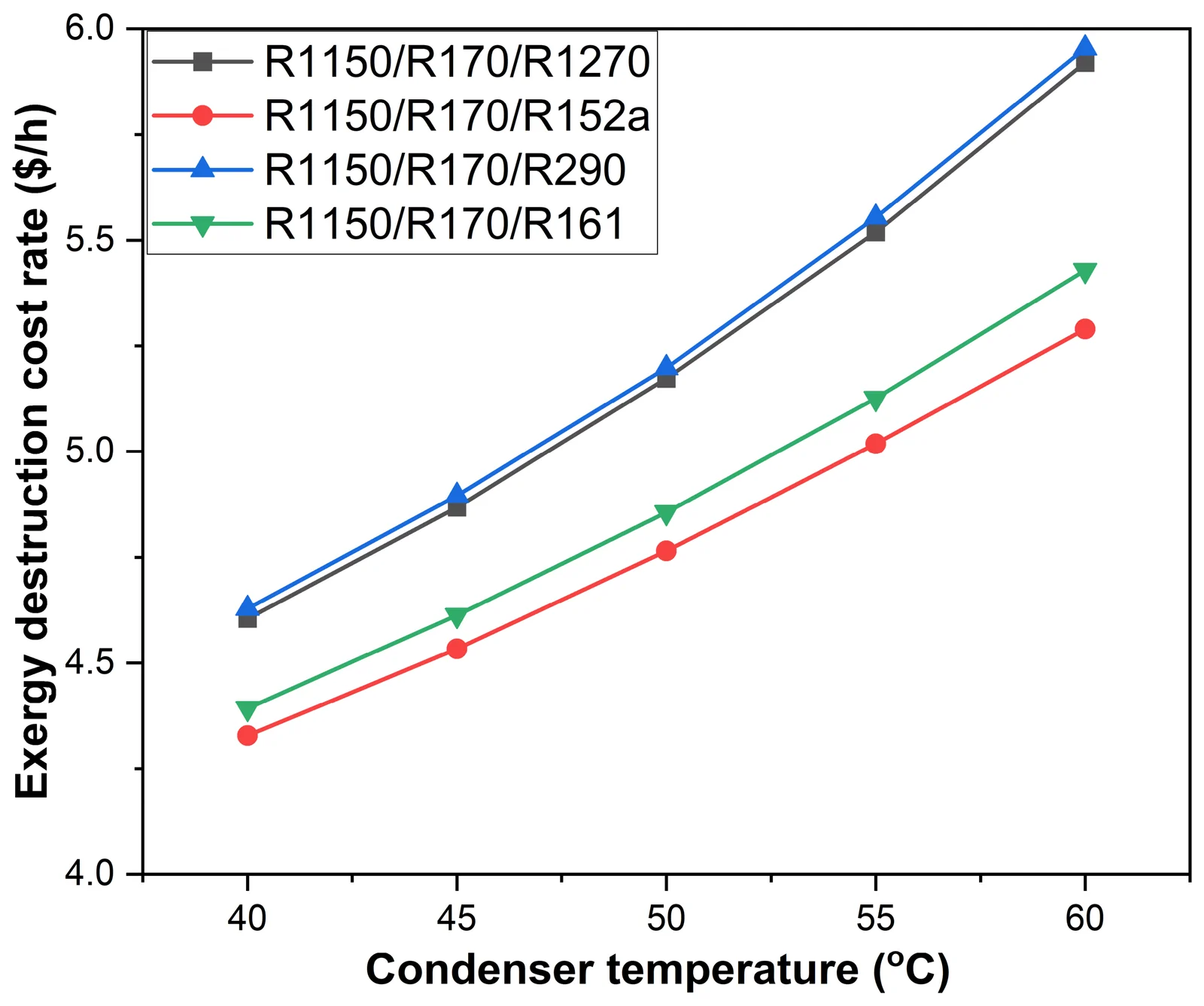
Alteration of exergy destruction cost rate illustrating the sensitivity of the TSCRS to condenser temperature.

The variation in ${\dot{C}}_{D,sys}$ with the condenser temperature for the investigated refrigerant combinations is presented in Figure 11 at a fixed evaporator temperature of −100 °C. As the condenser temperature increases from 40 °C to 60 °C, ${\dot{C}}_{D,sys}$ increases for all the combinations. Increasing the temperature of the condenser increases the pressure of the condensation process, thereby increasing the pressure ratio of the compressors and the corresponding power consumed. This increases the exergy destruction rate of the compressors and the heat exchangers, thereby increasing the ${\dot{C}}_{D,sys}$ of the system. Among the combinations, R1150/R170/R290 indicates the maximum ${\dot{C}}_{D,sys}$ over the entire range of temperature. As the condenser temperature increases, ${\dot{C}}_{D,sys}$ increases from 4.629 $/h to 5.954 $/h. Similar behavior is also observed for R1150/R170/R1270, where the ${\dot{C}}_{D,sys}$ increases from 4.605 $/h to 5.920 $/h. On the other hand, R1150/R170/R152a indicates the minimum ${\dot{C}}_{D,sys}$ for the entire range of the temperature, which increases from 4.328 $/h to 5.290 $/h. For R1150/R170/R161, ${\dot{C}}_{D,sys}$ increases from 4.393 $/h to 5.429 $/h.

**Figure 12:**
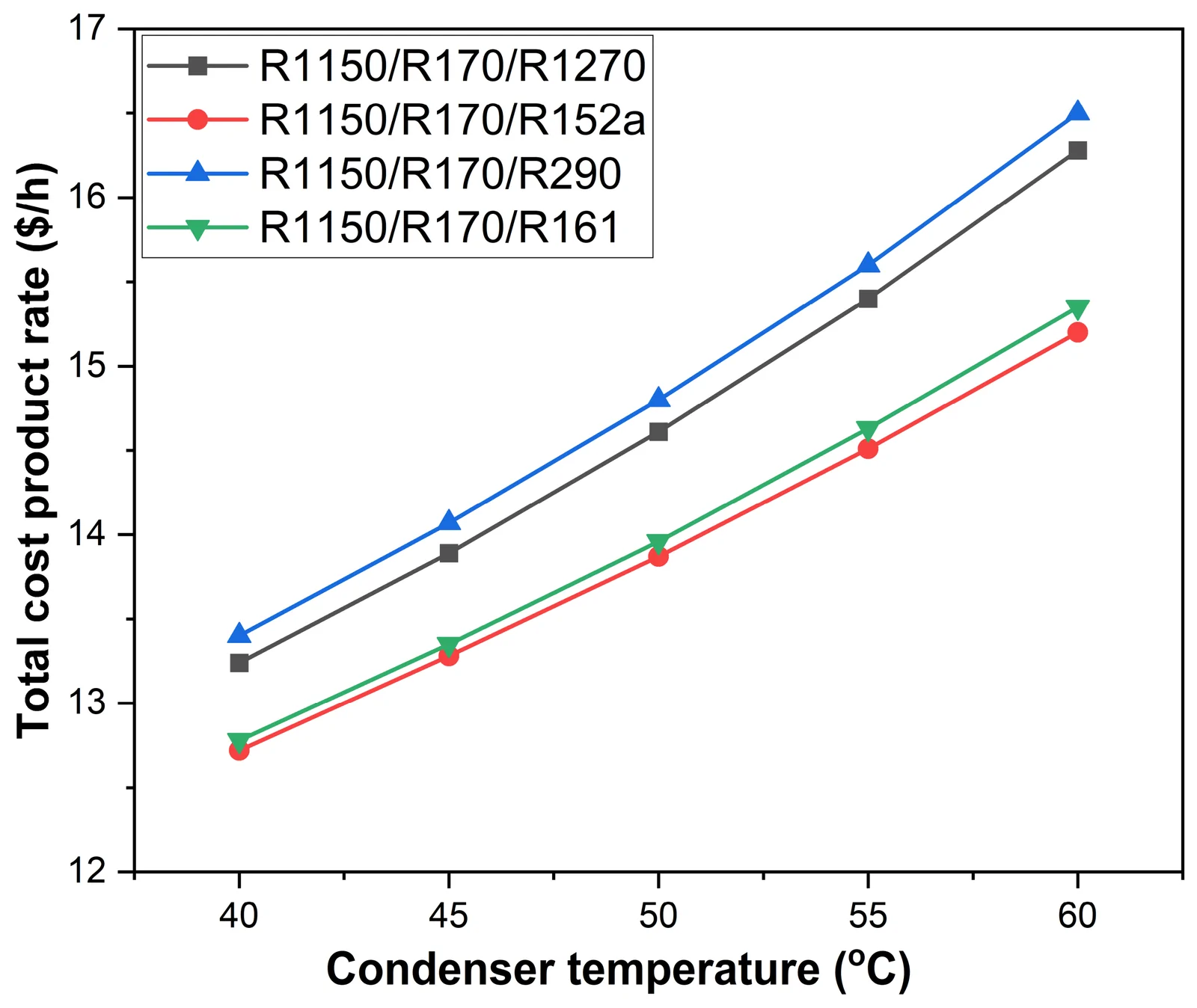
Alteration of the total cost product rate illustrating the sensitivity of the TSCRS to condenser temperature.

The variation in ${\dot{C}}_{P,sys}$ with the condenser temperature for the different refrigerant combinations is presented in Figure 12 at a fixed evaporator temperature of −100 °C. As the condenser temperature increases, the compression ratio of the system is also elevated, thereby increasing the power consumption of the compressor. Since the fuel exergy cost rate is directly related to the power consumption of the compressor, this leads to a high fuel exergy cost rate. As a result, ${\dot{C}}_{P,sys}$ is elevated. For the lowest possible condenser temperature of 40 °C, R1150/R170/R152a and R1150/R170/R161 have the lowest ${\dot{C}}_{P,sys}$ at 12.72 $/h and 12.78 $/h, respectively, whereas ${\dot{C}}_{P,sys}$ values are observed for R1150/R170/R1270 and R1150/R170/R290 at 13.24 $/h and 13.40 $/h, respectively. When the temperature is raised, ${\dot{C}}_{P,sys}$ increases almost linearly for all the combinations, but the rate of increase is not the same for all the refrigerant combinations. For example, at a temperature of 60 °C, ${\dot{C}}_{P,sys}$ values are 15.20 $/h for R1150/R170/R152a, 15.35 $/h for R1150/R170/R161, 16.28 $/h for R1150/R170/R1270, and 16.50 $/h for R1150/R170/R290.

**Figure 13:**
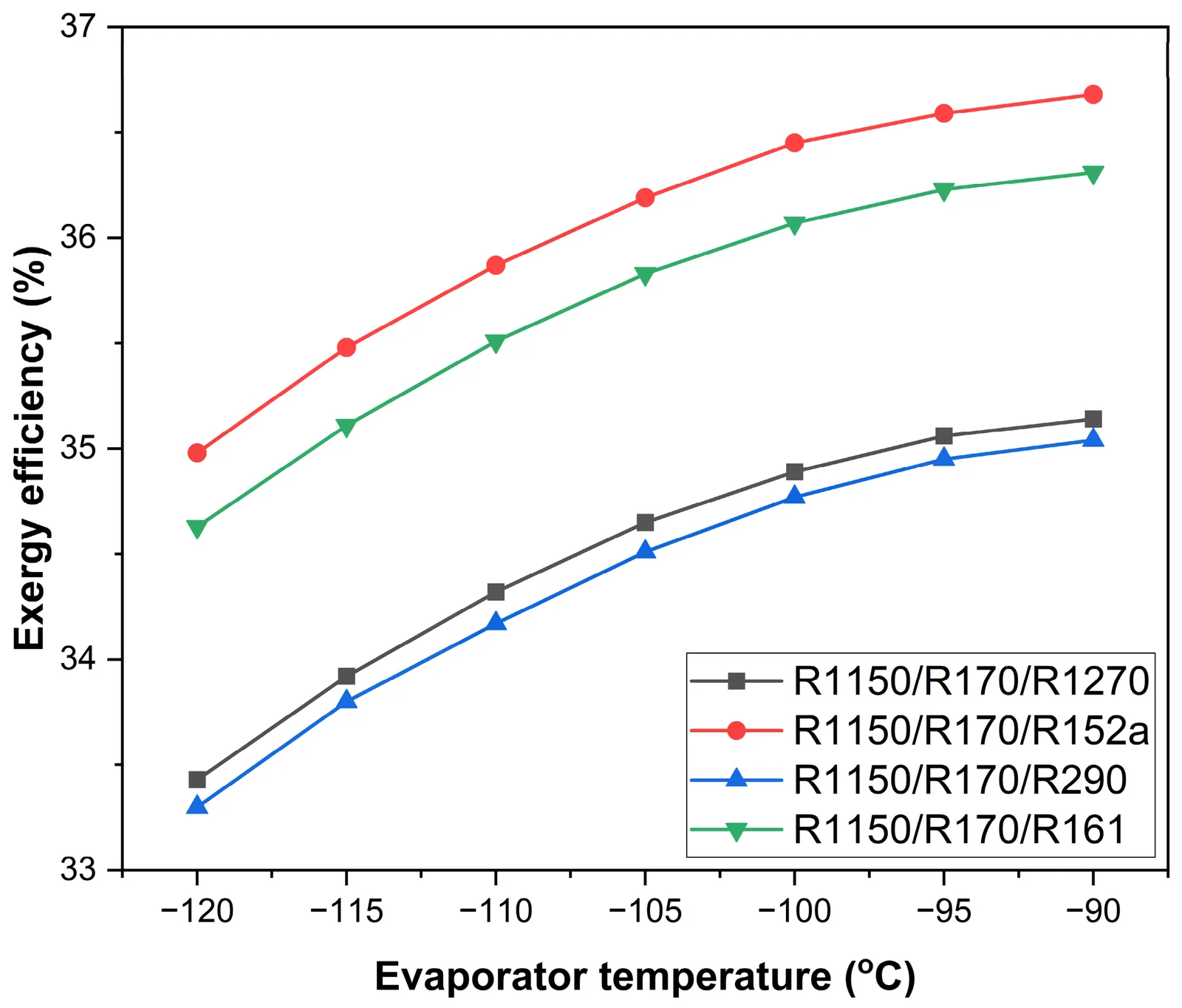
Alteration of the exergy efficiency illustrating the sensitivity of the TSCRS to evaporator temperature.

The variation in exergy efficiency with the change in temperature of the condenser for the different combinations of refrigerants is shown in Figure 13 at a fixed evaporator temperature of −100 °C. Higher temperatures in the evaporator result in greater exergy efficiency since the amount of work required to compress increases, leading to less irreversibility. With less exergy being lost in the process due to its smaller proportion in the total exergy input, more exergy is converted into the desirable refrigeration effect, improving the exergy efficiency. With the increase in evaporator temperature from −120 °C to −90 °C, exergy efficiency increases from 33.43% to 35.14% for R1150/R170/R1270, from 34.98% to 36.68% for R1150/R170/R152a, from 33.30% to 35.04% for R1150/R170/R290, and from 34.63% to 36.31% for R1150/R170/R161. Among the different combinations of refrigerant, R1150/R170/R152a yields the highest exergy efficiency, while R1150/R170/R290 yields the lowest exergy efficiency over the entire range of evaporator temperature.

**Figure 14:**
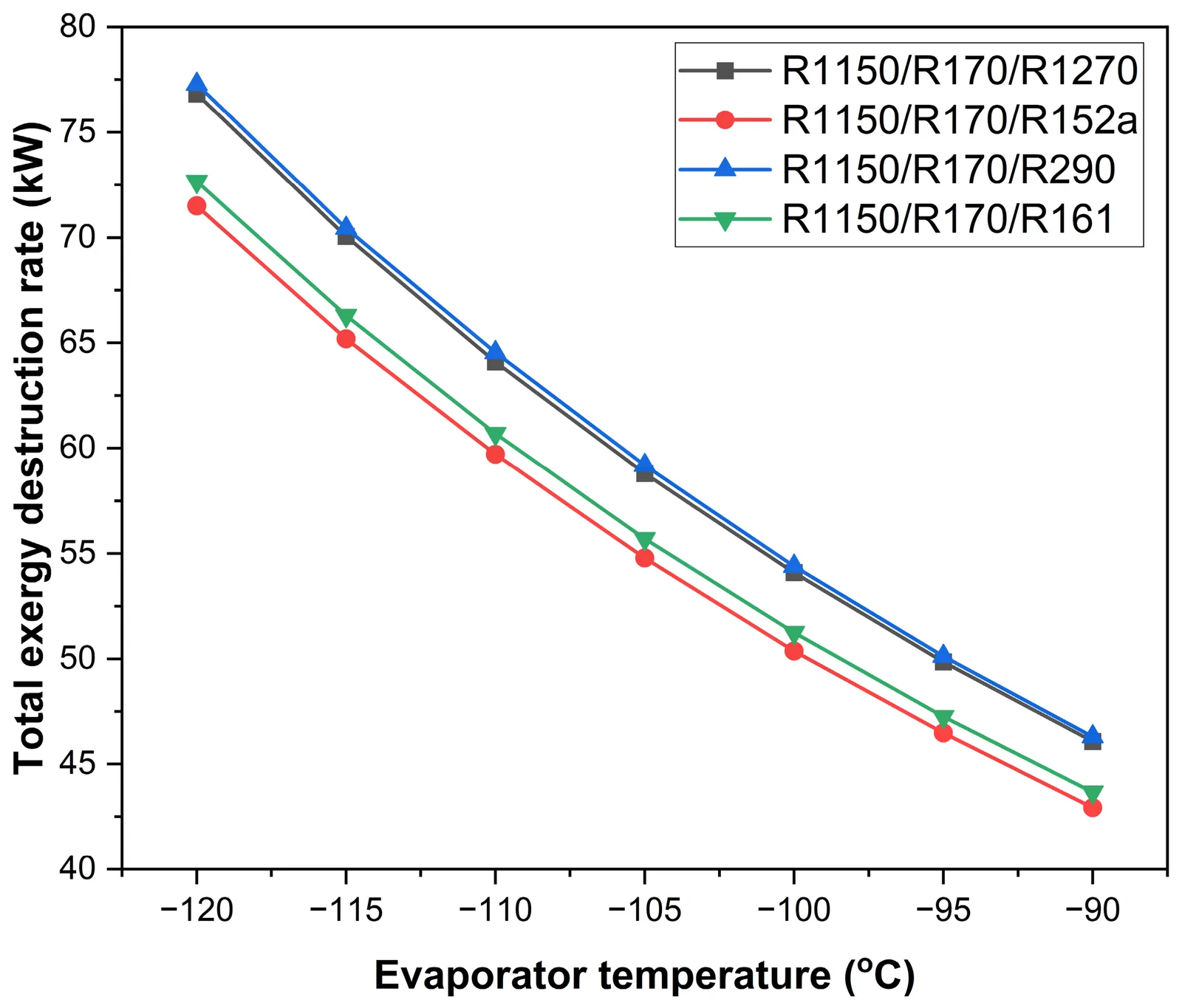
Alteration of the total exergy destruction rate illustrating the sensitivity of the TSCRS to evaporator temperature.

The variation in the total exergy destruction rate with the change in the temperature of the evaporator for the different combinations of refrigerants is shown in Figure 14 at a fixed condenser temperature of 45 °C. As the evaporator temperature increases from −120 °C to −90 °C, the exergy destruction reduces for each of the given refrigerant combinations. The observed decrease in the total exergy destruction for the system can be attributed to the decrease in the temperature lift of the system. With an increase in evaporator temperature, evaporation pressure is increased, which causes a reduction in the compressor pressure ratio and power consumption. This leads to a reduction in compressor irreversibility and entropy generation, leading to total exergy destruction. The exergy destruction for R1150/R170/R1270 reduces from 76.81 kW to 46.08 kW, from 71.50 kW to 42.93 kW for R1150/R170/R152a, from 77.28 kW to 46.30 kW for R1150/R170/R290, and from 72.66 kW to 43.67 kW for R1150/R170/R161. Among all the combinations, it has been found that R1150/R170/R290 has the highest total exergy destruction, and R1150/R170/R152a has the lowest total exergy destruction for all evaporator temperatures.

**Figure 15:**
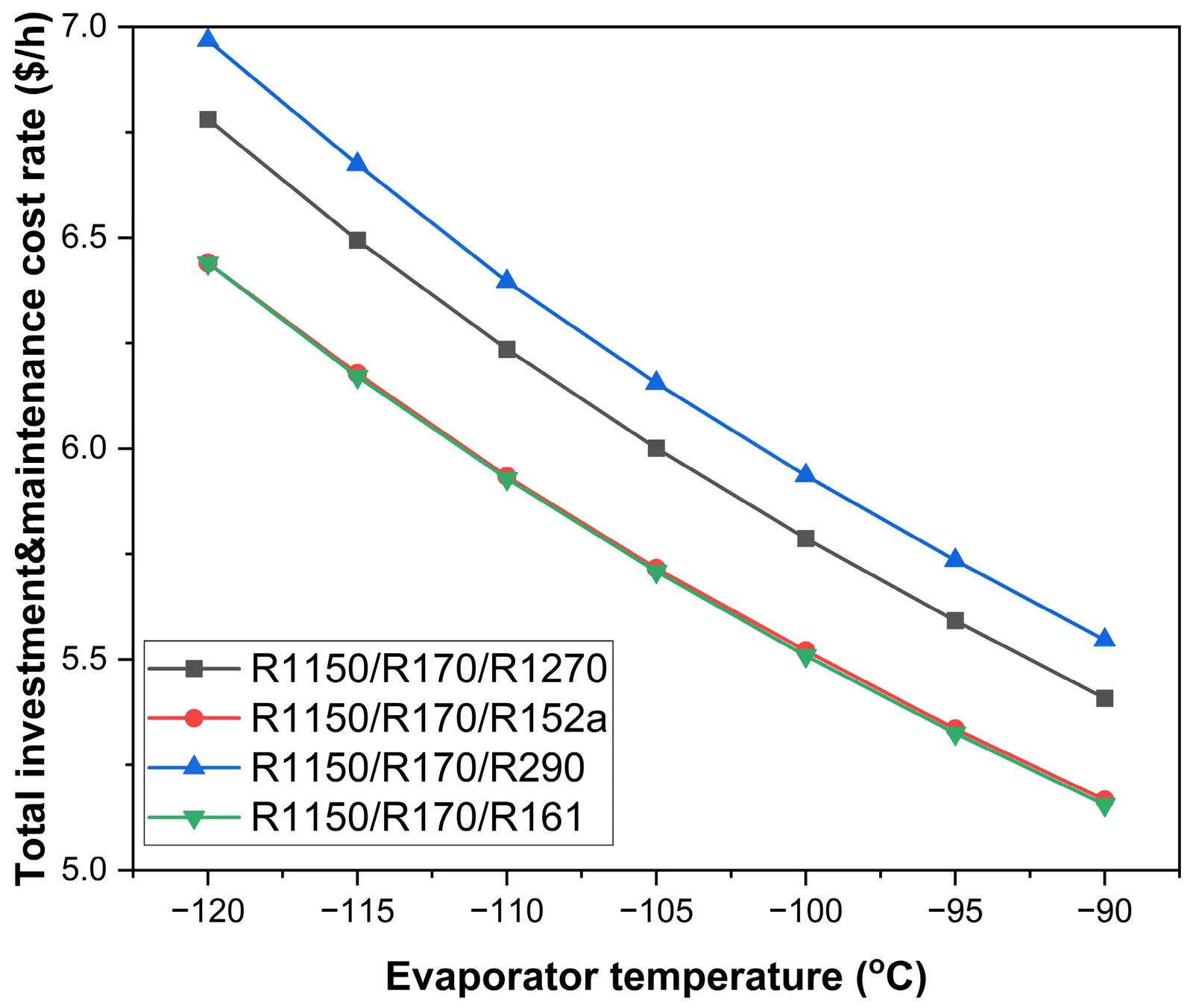
Alteration of the total investment & maintenance cost rate illustrating the sensitivity of the TSCRS to evaporator temperature.

The variation in ${\dot{Z}}_{sys}^{CI\&OM}$ with the change in the temperature of the evaporator for the different combinations of refrigerants is shown in Figure 15 at a fixed condenser temperature of 45 °C. The decrease in values can be attributed to the fact that the power requirements of the compressors decrease as the evaporator temperature increases. This is because the pressure ratio to which the compressors are subjected decreases as the evaporator temperature increases. This also reduces the thermal load on the components in the system and, therefore, on the heat exchangers and compressors. This leads to a decrease in ${\dot{Z}}_{sys}^{CI\&OM}$. The highest values are observed for R1150/R170/R290, ranging from 6.968 $/h to 5.546 $/h. A slightly lower range of values, from 6.781 $/h to 5.408 $/h, can be observed for R1150/R170/R1270. These values are still significantly higher than those of R152a and R161. The lowest ${\dot{Z}}_{sys}^{CI\&OM}$ values are observed for R1150/R170/R152a and R1150/R170/R161, which present very similar behavior over the entire range of investigated temperatures, reducing from 6.44 $/h to 5.167 $/h and from 6.44 $/h to 5.156 $/h, respectively. These results confirm that R152a- and R161-containing refrigerant combinations present better economic characteristics, whereas the R290-based combination requires the highest ${\dot{Z}}_{sys}^{CI\&OM}$.

**Figure 16:**
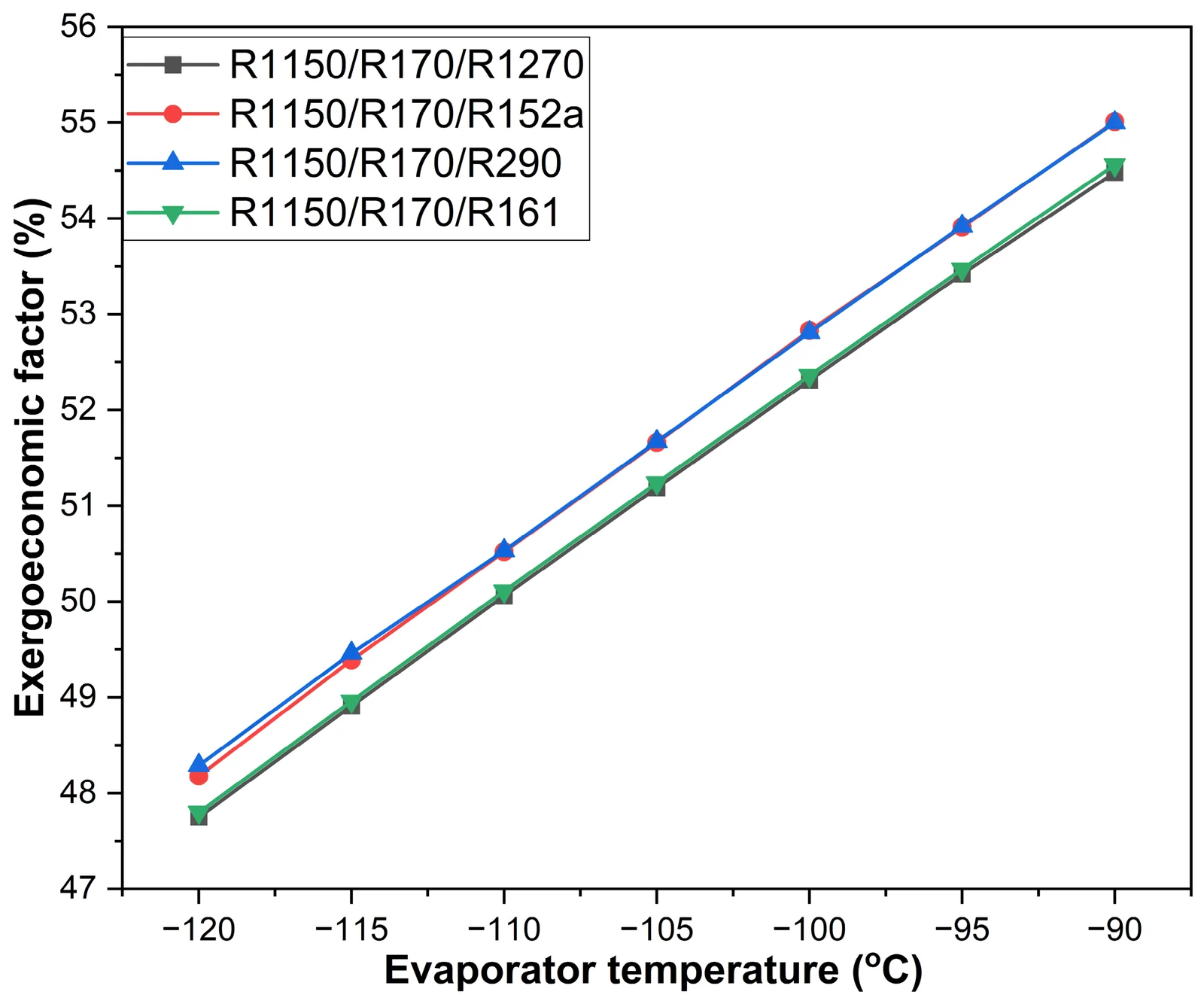
Alteration of the exergoeconomic factor illustrating the sensitivity of the TSCRS to evaporator temperature.

The variation in the exergoeconomic factor with the change in the temperature of the evaporator for the different combinations of refrigerants is shown in Figure 16 at a fixed condenser temperature of 45 °C. The increase in the exergoeconomic factor due to the enhancement in the evaporator temperature can be mainly attributed to the decline in the cost of exergy destruction. This is due to the decrease in the compression ratio and the power required to compress the refrigerant, which in turn leads to a decrease in the exergy destruction of the system. This results in a reduction in the exergy destruction cost rate compared to the investment and operating-maintenance cost rate, thereby improving the exergoeconomic factor. As the evaporator temperature rises from −120 °C to −90 °C, the exergoeconomic factor for R1150/R170/R1270 increases from 47.75% to 54.48%; for R1150/R170/R152a, it increases from 48.18% to 55.01%, for R1150/R170/R290 it improves from 48.29% to 55.00%, and for R1150/R170/R161 it rises from 47.80% to 54.56%. Throughout the temperature range, R1150/R170/R152a and R1150/R170/R290 consistently achieve the highest exergoeconomic factor values, while R1150/R170/R1270 and R1150/R170/R161 exhibit slightly lower levels, although the differences between the combinations remain relatively small.

**Figure 17:**
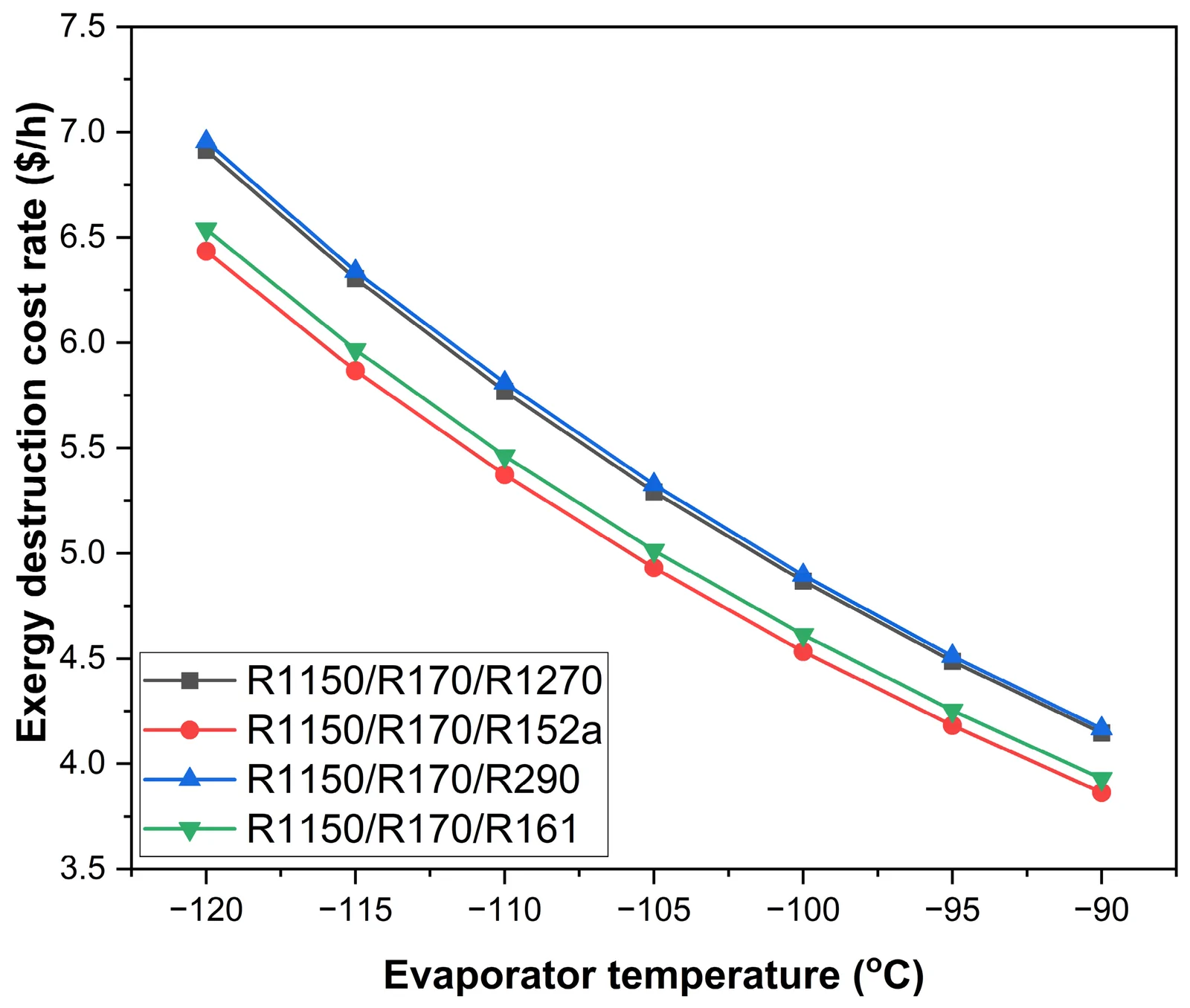
Alteration of the exergy destruction cost rate illustrating the sensitivity of the TSCRS to evaporator temperature.

The variation in ${\dot{C}}_{D,sys}$ with the change in the temperature of the evaporator for the different combinations of refrigerants is shown in Figure 17 at a fixed condenser temperature of 45 °C. The decrease in ${\dot{C}}_{D,sys}$ due to the increase in the evaporator temperature is mainly due to a decrease in the temperature lift of the system. With the increase in the evaporator temperature, the compressors operate at a lower pressure ratio, leading to a reduction in the compressors’ power requirements and therefore a reduction in the ${\dot{C}}_{D,sys}$. At the lowest evaporator temperature, the ${\dot{C}}_{D,sys}$ levels are observed for R1150/R170/R290 (6.955$/h) and R1150/R170/R1270 (6.913$/h), while R1150/R170/R152a (6.435$/h) and R1150/R170/R161 (6.539$/h) exhibit relatively low values. As the evaporator temperature increases to −90 °C, ${\dot{C}}_{D,sys}$ decreases to 4.167$/h, 4.147$/h, 3.864$/h, and 3.93$/h for R1150/R170/R290, R1150/R170/R1270, R1150/R170/R152a, and R1150/R170/R161, respectively. This comparison shows that R1150/R170/R152a maintains the lowest ${\dot{C}}_{D,sys}$ throughout the examined temperature range, while R1150/R170/R290 is the least preferred option with consistently higher values.

**Figure 18:**
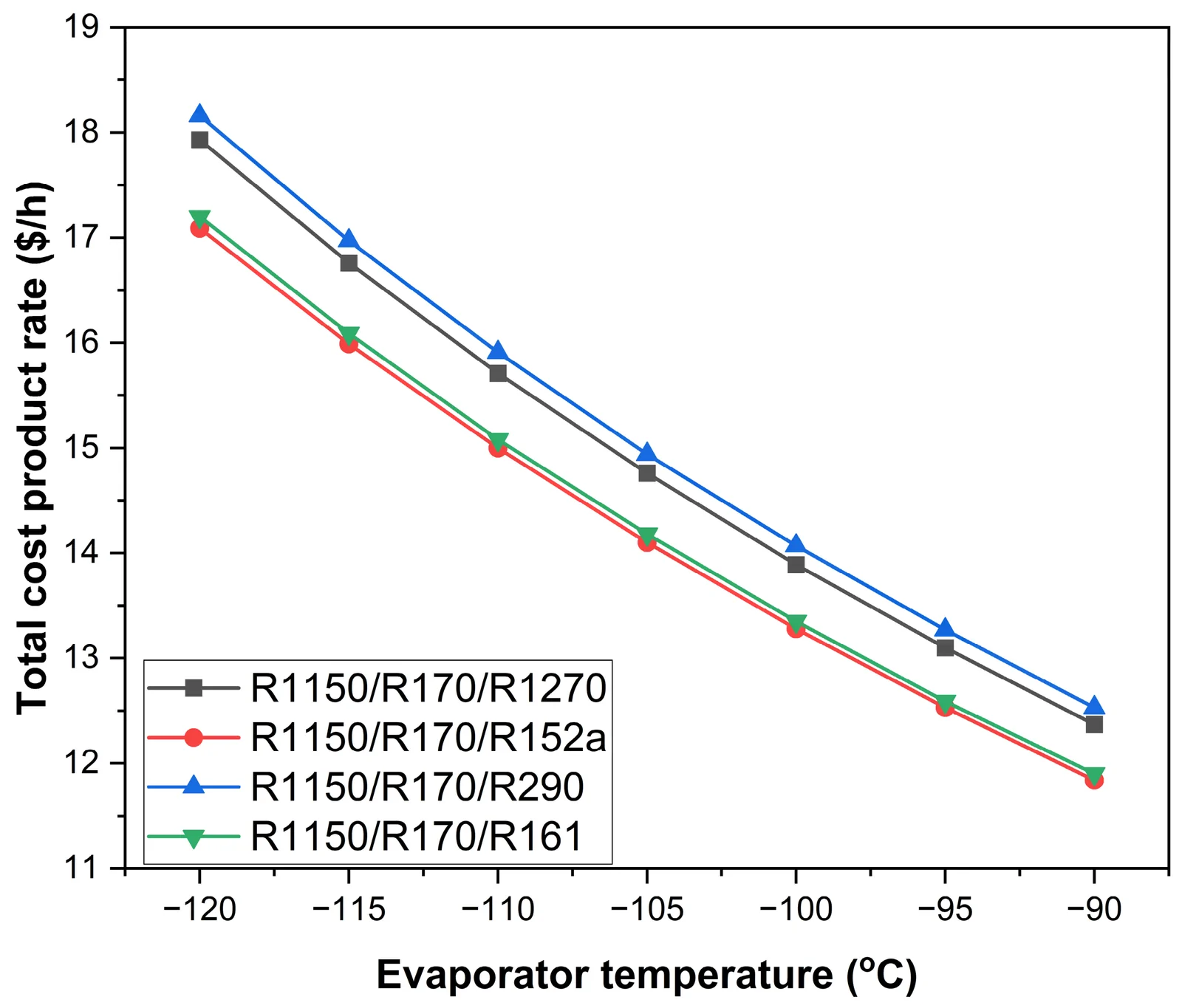
Alteration of the total product cost rate illustrating the sensitivity of the TSCRS to evaporator temperature.

The variation in ${\dot{C}}_{P,sys}$ with the change in the temperature of the evaporator for the different combinations of refrigerants is shown in Figure 18 at a fixed condenser temperature of 45 °C. As the evaporator temperature increases, the required compression ratio decreases, which leads to a reduction in the compressor’s power consumption. Since the fuel exergy cost rate is related to the compressor power consumption, the fuel exergy cost rate of the system decreases. This term constitutes a large portion of the ${\dot{C}}_{P,sys}$ value. Therefore, as the evaporator temperature increases, ${\dot{C}}_{P,sys}$ decreases. The ${\dot{C}}_{P,sys}$ values are reduced from 17.93$/h to 12.37$/h for R1150/R170/R1270, from 17.09$/h to 11.84$/h for R1150/R170/R152a, from 18.16$/h to 12.53$/h for R1150/R170/R290, and from 17.20$/h to 11.90$/h for R1150/R170/R161. At the lowest evaporator temperature, R1150/R170/R290 exhibits the highest ${\dot{C}}_{P,sys}$, exceeding R1150/R170/R152a by approximately 1.07$/h, while R1150/R170/R1270 and R1150/R170/R161 show higher values of 0.84$/h and 0.11$/h, respectively, than the R152a-based combination. A similar trend is observed at an evaporator temperature of −90 °C, where ${\dot{C}}_{P,sys}$ reaches a maximum of 12.53 $/h for R1150/R170/R290, 12.37 $/h for R1150/R170/R1270, 11.90 $/h for R1150/R170/R161, and 11.84 $/h for R1150/R170/R152a. It is clear that the combination of R1150/R170/R152a has the lowest ${\dot{C}}_{P,sys}$, while the combination of R1150/R170/R290 has the highest ${\dot{C}}_{P,sys}$ throughout the investigated evaporator temperatures.

The obtained results demonstrate that refrigerant selection has a substantial impact on exergy destruction, cost distribution, and overall system performance, highlighting its importance in the design and optimization of efficient TSCRSs. The outcomes indicate that the refrigerant choice is an important factor in determining the performance of the TSCRSs. As such, the results obtained in this study can be used as a guide for future studies focusing on the screening and optimization of sustainable refrigeration systems.

## 4. Conclusions

This study presents a complete exergy and exergoeconomic analysis and optimization of TSCRS using different refrigerant combinations with low GWP. Component-wise and parametric analyses are performed to assess the influence of key parameters on component and system performance. The importance of the current work stems from the fact that it presents an exergoeconomic approach for evaluating and optimizing TSCRSs using the SPECO method. The findings contribute to the knowledge about the impact of the choice of refrigerant on exergy destruction, costing, and system performance. The main findings obtained under the operating conditions are summarized as follows:

- The most favorable component-wise thermodynamic and exergoeconomic behavior is generally obtained with R1150/R170/R152a and R1150/R170/R161, whereas R1150/R170/R290 tends to exhibit the least favorable distribution. Specifically, the highest relative exergy destruction ratio is observed in TV-3 for R1150/R170/R1270 at 24.71%, while the lowest value occurs in TV-1 for R1150/R170/R1270 at 3.83%. In terms of the exergy destruction ratio, the highest value is obtained in the HTC compressor for R1150/R170/R1270 at 14.92%, whereas the lowest value occurs in the LTC compressor for R1150/R170/R1270 at 2.31%, indicating that the major thermodynamic irreversibilities are mainly concentrated in the compression and expansion processes of the system.
- The evaporator exhibits the highest Z_k_ + Ċ_L,k_ for R1150/R170/R290 at 24.73 $/h, whereas the lowest value is obtained for R1150/R170/R161 at 23.69 $/h. Likewise, the condenser reaches the highest exergoeconomic factor for R1150/R170/R152a at 14.24%, while the throttling valves exhibit the lowest values for all refrigerant combinations, with the minimum occurring in TV-1 for R1150/R170/R290 at 0.170%.
- Ċ_D,k_ is the dominant cost rate term in almost all system components, while Z_k_ becomes more significant in the evaporator, compressors and condenser, and Ċ_L,k_ is significant only in the condenser.
- R1150/R170/R152a consistently provides the highest exergy efficiency and the lowest total exergy destruction rate. Compared with R1150/R170/R290, it improves exergy efficiency and total exergy destruction by up to 5.05% and 7.50% under evaporator temperature variations and by up to 7.84% and 11.14% under condenser temperature variations, respectively.
- R1150/R170/R161 yields the lowest Ż_sys_ values within the investigated temperature ranges. Compared with R1150/R170/R290, Ż_sys_ is reduced by up to 7.58% for evaporator temperature variations and up to 8.77% for condenser temperature variations.
- R1150/R170/R152a achieves the lowest Ċ_D,sys_ values over the entire temperature ranges. Compared with R1150/R170/R290, this combination reduces Ċ_D,sys_ by up to 7.51% for evaporator temperature variations and up to 11.15% for condenser temperature variations.
- The lowest Ċ_p,sys_ values are consistently obtained with R1150/R170/R152a, whereas the highest values are produced by R1150/R170/R290. Ċ_p,sys_ decreases by up to 5.89% under evaporator temperature variations and by up to 7.88% under condenser temperature variations.
- The highest exergoeconomic factor is generally achieved with R1150/R170/R152a or R1150/R170/R290, while the lowest values are observed for R1150/R170/R1270. The improvement in the exergoeconomic factor reaches up to 1.13% for evaporator temperature variations and up to 2.03% for condenser temperature variations.

The presented methodology contributes to the field of sustainable refrigeration in general through the provision of assistance in future research in the area of refrigerant choice and optimization, thereby increasing thermodynamic and exergoeconomic performance.

The findings from this study can have practical applications in the optimization of TSCRSs, emphasizing the importance of refrigerants used in relation to the amount of energy destruction, cost sharing, and system efficiency. The framework presented may be helpful to the research community in finding more efficient and economically feasible refrigeration systems.

## 5. Limitations and Future Research Directions

Although the present research work presents a detailed exergy and exergoeconomic analysis of the TSCRS, there are a few limitations that should be acknowledged. The present analysis is performed under steady-state operating conditions, neglecting the transient effects. The exergoeconomic analysis is based on the general correlations, which may vary according to the actual market conditions due to the variation of the interest rate and electricity costs. Moreover, it should be noted that the present research work focuses only on the exergy and exergoeconomic analysis, whereas the environmental impact is not considered. These limitations can be overcome by future studies that may include experimental studies to validate the performance of the TSCRS under actual operating conditions. Furthermore, more detailed component modeling can be considered with special emphasis on compressor and heat exchanger modeling to improve the accuracy of performance predictions of the system. Future studies may also include environmental impact assessment techniques like Total Equivalent Warming Impact or Life Cycle Climate Performance to assess the environmental implications of different combinations of refrigerants. In addition to that, the use of multi-objective optimization techniques to optimize thermodynamic performance, economic performance, and environmental impact may provide a more comprehensive evaluation of the performance of the system.

## Figures and Tables

**Table 2:** Properties of refrigerants used in this study for TSCRS [27,52,53].

Cycle	Refrigerant	Molecular Mass (kg/kmol)	NBP (°C)	Critical Temperature (°C)	Critical Pressure (bar)	ASHRAE Safety Group	ODP	GWP (100 Years)
LTC	R-1150	28.05	−103.77	9.2	50.4	A3	0	4
MTC	R-170	30.1	−88.58	32.2	48.7	A3	0	5.5
HTC	R-290	44.1	−42.1	96.7	42.5	A3	0	<1
	R-1270	42.1	−47.6	92.4	46.7	A3	0	<1
	R-152a	66.05	−24.02	113.3	45.2	A2	0	138
	R-161	48.06	−37.55	102.2	50.9	A3	0	4

**Table 3:** Design parameters and values of the presented model [3,26,40,54].

Dead state temperature, *T_o_*	25 °C
Dead state pressure, *P_o_*	101.325 kPa
Cooling effect, $\dot{Q}$*_evap_*	50 kW
Evaporator temperature, *T_evap_*	−120 °C to −90 °C
Condenser temperature, *T_cond_*	40 °C to 60 °C
Temperature difference in CHXs, Δ*T*	5 °C
Isentropic efficiency compressors, *η_isen_*	0.8
Mechanical efficiency of compressors, *η_mech_*	0.9
Electrical efficiency of compressors, *η_elect_*	0.95
Temperature difference between air at the inlet and outlet for evaporator and condenser	10 °C
Maintenance factor, *ϕ*	1.06
Interest rate, *i*	14%
Plant lifetime, *n*	15 years
Annual operational hours, *N*	4266 h
Electrical power cost, *c_elect_*	0.09 $/kWh
Overall heat transfer coefficient of evaporator, *U_evap_*	0.03 kW/m^2^K
Overall heat transfer coefficient of condenser, *U_cond_*	0.04 kW/m^2^K
Overall heat transfer coefficient of CHXs, *U_CHX_*	1 kW/m^2^K

**Table 5:** Cost functions of all equipment forming the TSCRS [61,62,63].

Component	Cost Function ($)	Reference Year and CEPCI	Base Year and CEPCI	$\frac{{CEPCI}_{2025}}{{CEPCI}_{ref}}$
Evaporator	$Z_{evap}=16,000{\left(\frac{A_{LTC,evap}}{100}\right)}^{0.6}$	2000—389.5	2025—819.3	2.10
Condenser	$Z_{cond}=8000{\left(\frac{A_{\mathrm{HTC},\mathrm{cond}}}{100}\right)}^{0.6}$	2000—394.1	2025—819.3	2.08
LTC compressor	$Z_{LTC,comp}=12,000\left(\frac{{\dot{W}}_{LTC,comp}}{100}\right){\left(\frac{\eta_{LTC,comp}}{0.9-\eta_{LTC,comp}}\right)}^{0.5}$	1998—389.5	2025—819.3	2.10
MTC compressor	$Z_{MTC,comp}=12,000\left(\frac{{\dot{W}}_{MTC,comp}}{100}\right){\left(\frac{\eta_{MTC,comp}}{0.9-\eta_{MTC,comp}}\right)}^{0.5}$	1998—389.5	2025—819.3	2.10
HTC compressor	$Z_{HTC,comp}=12,000\left(\frac{{\dot{W}}_{HTC,comp}}{100}\right){\left(\frac{\eta_{HTC,comp}}{0.9-\eta_{HTC,comp}}\right)}^{0.5}$	1998—389.5	2025—819.3	2.10
LTC throttle valve	700	2019—576.1	2025—819.3	1.42
MTC throttle valve	700	2019—576.1	2025—819.3	1.42
HTC throttle valve	700	2019—576.1	2025—819.3	1.42
CHX-1	$Z_{CHX\text{-}1}=12,000{\left(\frac{A_{\mathrm{CHX}\text{-}1}}{100}\right)}^{0.6}$	2000—394.1	2025—819.3	2.08
CHX-2	$Z_{CHX\text{-}2}=12,000{\left(\frac{A_{CHX\text{-}2}}{100}\right)}^{0.6}$	2000—394.1	2025—819.3	2.08

**Table 6:** Cost functions of all equipment forming the TSCRS [64].

Component	Cost Balance Equations	Auxiliary Equations
Evaporator	${\dot{C}}_{1}+{\dot{C}}_{14}={\dot{C}}_{4}+{\dot{C}}_{13}+{\dot{Z}}_{evap}$	$c_{1}=c_{4}$ $c_{13}=0$
Condenser	${\dot{C}}_{7}+{\dot{C}}_{10}={\dot{C}}_{6}+{\dot{C}}_{9}+{\dot{Z}}_{cond}$	$c_{10}=c_{11}$ $c_{15}=0$
LTC compressor	${\dot{C}}_{2}={\dot{C}}_{1}+{\dot{C}}_{w,LTC,comp}+{\dot{Z}}_{LTC,comp}$	$c_{elect}=0.09\;\$/kWh$ ${\dot{C}}_{w,LTC,comp}=c_{elect}{\dot{W}}_{LTC,comp}$
MTC compressor	${\dot{C}}_{6}={\dot{C}}_{5}+{\dot{C}}_{w,MTC,comp}+{\dot{Z}}_{MTC,comp}$	$c_{elect}=0.09\;\$/kWh$ ${\dot{C}}_{w,MTC,comp}=c_{elect}{\dot{W}}_{MTC,comp}$
HTC compressor	${\dot{C}}_{10}={\dot{C}}_{9}+{\dot{C}}_{w,HTC,comp}+{\dot{Z}}_{HTC,comp}$	$c_{elect}=0.09\;\$/kWh$ ${\dot{C}}_{w,HTC,comp}=c_{elect}{\dot{W}}_{HTC,comp}$
LTC throttle valve	${\dot{C}}_{4}={\dot{C}}_{3}+{\dot{Z}}_{LTC,tv}$	-
MTC throttle valve	${\dot{C}}_{8}={\dot{C}}_{7}+{\dot{Z}}_{MTC,tv}$	-
HTC throttle valve	${\dot{C}}_{12}={\dot{C}}_{11}+{\dot{Z}}_{HTC,tv}$	-
CHX-1	${\dot{C}}_{5}+{\dot{C}}_{3}={\dot{C}}_{2}+{\dot{C}}_{8}+{\dot{Z}}_{CHX\text{-}1}$	$c_{5}=c_{8}$
CHX-2	${\dot{C}}_{9}+{\dot{C}}_{7}={\dot{C}}_{6}+{\dot{C}}_{12}+{\dot{Z}}_{CHX\text{-}2}$	$c_{9}=c_{12}$

Note that to express the cost rates of exergy flows in $/h, the unit exergy costs must be converted from $/GJ into $/kWh. This conversion is carried out using 1 GJ = 277.78 kWh (or the multiplier 0.0036).

**Table 7:** Refrigerating effect and COP validation of VCRS against the experimental study of Ma and Zhao (2008) [67].

T_evap_ (°C)	*Refrigerating Effect (kJ/kg) and **COP				Relative Deviation (%)
	Experimental Setup [67]	Simulation Model [26]	Simulation Model [54]	Simulation Model (This Study)	
−15	146.39 */1.61 **	145.62 */1.63 **	-/1.63 **	145.85/1.625	0.37/0.93
−10	149.38 */2.23 **	148.85 */2.25 **	-/2.25 **	148.93/2.249	0.30/0.85
−5	152.49 */3.02 **	152.04 */3.04 **	-/3.04 **	152.28/3.036	0.14/0.53

**Table 8:** COP validation of CRS against the experimental study of Dopazo and Fernández-Seara (2011) [68].

LTC Condenser Temperature (°C)	Experimental Setup [68]	Simulation Model (This Study)	Relative Deviation (%)
−17	0.95	0.984	3.58
−15	0.97	0.958	1.24
−12.5	0.91	0.942	3.52
−10	0.89	0.914	2.70

**Table 9:** Energy and exergy parameter validation of TSCRS against the theoretical study of Nabil et al. (2023) [26].

Author/Parameter	[26]	This Study	Relative Deviation (%)
Condenser load (kW)	24.97	24.91	0.280
Total compressor work (kW)	17.51	17.48	0.171
COP	0.571	0.568	0.525
Exergy efficiency (%)	41.07	40.95	0.292

**Table 10:** COP validation of TSCRS against the theoretical study of Sun et al. (2019) [27].

LTC Condenser Temperature (°C)	Simulation Model [27]	Simulation Model [42]	Simulation Model [30]	Simulation Model (This Study)	Relative Deviation (%)
−85	0.5175	0.5129	0.5123	0.5169	0.116
−80	0.545	0.541	0.5403	0.5446	0.073
−75	0.5675	0.5623	0.5615	0.5668	0.123
−70	0.5825	0.5771	0.5764	0.5818	0.120
−65	0.59	0.5860	0.5852	0.5893	0.119
−60	0.5925	0.5891	0.5884	0.5919	0.101
−55	0.59	0.5866	0.5859	0.5891	0.153
−50	0.5825	0.5785	0.5779	0.5816	0.155
−45	0.5675	0.5650	0.5645	0.5669	0.106

**Table 11:** Total cost rate validation of TSCRS against the theoretical study of Kayes et al. (2024) [54].

LTC Condenser Temperature (°C)	Simulation Model (Kayas et al., 2024) [54]	Simulation Model (This Study)	Relative Deviation (%)
−80	34,976 $/year	34,890 $/year	**−0.246**
−75	35,031 $/year	34,920 $/year	**−0.317**
−72	35,111 $/year	34,985 $/year	**−0.359**
−68	35,222 $/year	35,010 $/year	**−0.602**
−64	35,357 $/year	35,140 $/year	**−0.614**
−60	35,512 $/year	35,280 $/year	**−0.653**
−56	35,685 $/year	35,420 $/year	**−0.742**
−52	35,879 $/year	35,610 $/year	**−0.750**
−48	36,083 $/year	35,750 $/year	**−0.923**
−44	34,976 $/year	34,850 $/year	**−0.360**

**Table 12:** COP validation of TSCRS against the theoretical study of Johnson et al. (2017) [46].

Parameter	Simulation Model [46]	Simulation Model [22]	Simulation Model (This Study)	Relative Deviation (%)
COP_LT_	0.6501	0.65	0.6502	0.0031
COP_MT_	0.7721	0.7757	0.7739	0.23
COP_HT_	1.5218	1.5536	1.5382	1.078
COP	0.1155	0.1170	0.1162	0.61

**Table 13:** Thermodynamic and exergoeconomic properties of R1150/R170/R1270 for each state.

State	T (°C)	P (kPa)	h (kJ/kg)	s (kJ/kg/°C)	$\dot{m}$ (kg/s)	$\dot{E}$ (kW)	C ($/GJ)	$\dot{C}$ ($/h)
1	−100	126	−174.9	−0.8152	0.1321	9.072	72.73	2.375
2	6.074	732.3	−41.64	−0.7167	0.1321	22.8	53.31	4.375
3	−60.83	732.3	−553.4	−3.064	0.1321	47.63	68.73	11.78
4	−100	126	−553.4	−3.001	0.1321	45.17	72.73	11.83
5	−65.83	298.8	−156.1	−0.9113	0.2049	23.82	66.07	5.664
6	20.58	1422	−38.68	−0.8297	0.2049	42.89	54.39	8.398
7	−20	1422	−486	−2.573	0.2049	57.75	60.93	12.67
8	−65.83	298.8	−486	−2.503	0.2049	53.44	66.07	12.71
9	−25	256.3	−82.53	−0.4767	0.3918	23.67	58.76	5.008
10	75.51	1847	40.91	−0.4047	0.3918	63.63	45.85	10.5
11	45	1847	−316.5	−1.519	0.3918	53.75	45.85	8.873
12	−25	256.3	−316.5	−1.419	0.3918	42.14	58.76	8.913
13	30	101.3	303.4	6.875	13.91	0.5805	0	0
14	40	101.3	313.5	6.908	13.91	5.113	152.7	2.81
15	−85	101.3	187.7	6.396	4.961	136.1	0	0
16	−95	101.3	177.6	6.34	4.961	167.5	18.38	11.08

**Table 14:** Thermodynamic and exergoeconomic properties of R1150/R170/R152a for each state.

State	T (°C)	P (kPa)	h (kJ/kg)	s (kJ/kg/°C)	$\dot{m}$ (kg/s)	$\dot{E}$ (kW)	c ($/GJ)	$\dot{C}$ ($/h)
1	−100	126	−174.9	−0.8152	0.1291	8.866	71.42	2.28
2	−2.119	647.7	−52.65	−0.7222	0.1291	21.07	53.69	4.073
3	−64.17	647.7	−562.1	−3.104	0.1291	46.99	68.1	11.52
4	−100	126	−562.1	−3.052	0.1291	44.96	71.42	11.56
5	−69.17	259.1	−159.4	−0.8909	0.1895	20.24	64.87	4.726
6	12.92	1167	−47.21	−0.8108	0.1895	36.97	53.66	7.142
7	−26.91	1167	−506.6	−2.654	0.1895	54.03	60.55	11.78
8	−69.17	259.1	−506.6	−2.593	0.1895	50.61	64.87	11.82
9	−31.91	70.6	483.8	2.191	0.4299	−3.079	57.94	−0.6422
10	91.46	1038	598.1	2.255	0.4299	37.83	36.28	4.941
11	45	1038	281.3	1.272	0.4299	27.57	36.28	3.6
12	−31.91	70.6	281.3	1.351	0.4299	17.45	57.94	3.64
13	30	101.3	303.4	6.875	13.53	0.5645	0	0
14	40	101.3	313.5	6.908	13.53	4.973	132.3	2.368
15	−85	101.3	187.7	6.396	4.961	136.1	0	0
16	−95	101.3	177.6	6.34	4.961	167.5	18.1	10.91

**Table 15:** Thermodynamic and exergoeconomic properties of R1150/R170/R290 for each state.

State	T (°C)	P (kPa)	h (kJ/kg)	s (kJ/kg/°C)	$\dot{m}$ (kg/s)	$\dot{E}$ (kW)	c ($/GJ)	$\dot{C}$ ($/h)
1	−100	126	−174.9	−0.8152	0.1331	9.142	73.74	2.427
2	8.758	762.1	−38	−0.715	0.1331	23.39	53.42	4.498
3	−59.72	762.1	−550.4	−3.05	0.1331	47.86	69.47	11.97
4	−100	126	−550.4	−2.984	0.1331	45.24	73.74	12.01
5	−64.72	312.9	−155	−0.9179	0.209	24.93	67.18	6.028
6	21.74	1481	−37.86	−0.8368	0.209	44.35	55.17	8.809
7	−18.52	1481	−481.5	−2.556	0.209	58.77	61.88	13.09
8	−64.72	312.9	−481.5	−2.484	0.209	54.3	67.18	13.13
9	−23.52	215	547.8	2.405	0.4115	19.93	60.33	4.328
10	64.73	1534	663.6	2.475	0.4115	59.03	45.84	9.741
11	45	1534	322.5	1.407	0.4115	49.7	45.84	8.2
12	−23.52	215	322.5	1.503	0.4115	37.94	60.33	8.241
13	30	101.3	303.4	6.875	13.94	0.5818	0	0
14	40	101.3	313.5	6.908	13.94	5.125	154.8	2.856
15	−85	101.3	187.7	6.396	4.961	136.1	0	0
16	−95	101.3	177.6	6.34	4.961	167.5	18.6	11.21

**Table 16:** Thermodynamic and exergoeconomic properties of R1150/R170/R161 for each state.

State	T (°C)	P (kPa)	h (kJ/kg)	s (kJ/kg/°C)	$\dot{m}$ (kg/s)	$\dot{E}$ (kW)	c ($/GJ)	$\dot{C}$ ($/h)
1	−100	126	−174.9	−0.8152	0.1296	8.9	70.41	2.256
2	−0.7335	661.4	−50.79	−0.7213	0.1296	21.35	53.12	4.083
3	−63.61	661.4	−560.7	−3.098	0.1296	47.09	67.04	11.37
4	−100	126	−560.7	−3.043	0.1296	45	70.41	11.41
5	−68.61	265.5	−158.9	−0.8944	0.1916	20.77	63.61	4.757
6	13.92	1201	−46.17	−0.8142	0.1916	37.79	53.01	7.21
7	−25.93	1201	−503.8	−2.642	0.1916	54.55	59.29	11.64
8	−68.61	265.5	−503.8	−2.581	0.1916	51.02	63.61	11.68
9	−30.93	136.3	554.1	2.479	0.3488	7.674	55.98	1.547
10	97.78	1538	695.8	2.557	0.3488	48.97	40.63	7.162
11	45	1538	302.7	1.342	0.3488	38.17	40.63	5.583
12	−30.93	136.3	302.7	1.441	0.3488	27.9	55.98	5.623
13	30	101.3	303.4	6.875	13.62	0.5683	0	0
14	40	101.3	313.5	6.908	13.62	5.006	142.5	2.569
15	−85	101.3	187.7	6.396	4.961	136.1	0	0
16	−95	101.3	177.6	6.34	4.961	167.5	17.88	10.78

**Table 17:** Exergoeconomic cost rates for components under different refrigerant combinations.

Component	${\dot{Z}}_{k}$ ($/h)	${\dot{C}}_{D,k}$ ($/h)	${\dot{C}}_{L,k}$ ($/h)
CHX-1	0.354 *	19.93	-
	0.365 **	19.94	
	0.368 ***	20.15	
	0.356 ****	19.49	
CHX-2	0.354	17.33	-
	0.365	17.03	
	0.368	17.37	
	0.356	16.70	
LTC comp	0.373	6.959	-
	0.416	7.016	
	0.431	7.034	
	0.380	6.969	
MTC comp	0.502	7.084	-
	0.568	7.135	
	0.578	7.143	
	0.510	7.091	
HTC comp	1.161	7.493	-
	1.142	7.434	
	1.125	7.387	
	1.167	7.519	
TV-1	0.040	23.46	-
	0.040	23.46	
	0.040	23.64	
	0.040	23.06	
TV-2	0.040	20.42	-
	0.040	20.29	
	0.040	20.58	
	0.040	19.96	
TV-3	0.040	8.269	-
	0.040	12.94	
	0.040	12.60	
	0.040	10.69	
Cond	1.027	5.607	0.397
	1.180	7.576	0.408
	1.315	8.029	0.409
	0.990	6.012	0.399
Evap	1.63	22.38	-
	1.63	22.79	
	1.63	23.10	
	1.63	22.06	

* R1150/R170/R1270, ** R1150/R170/R152a, *** R1150/R170/R290, **** R1150/R170/R161.

**Table 18:** Cost dominance classification for each component.

Component	Investment Cost Contribution	Exergy Destruction Cost Contribution	Exergy Loss Cost Contribution
CHX-1	Low	Very high	–
CHX-2	Low	Very high	–
LTC compressor	Moderate	High	–
MTC compressor	Moderate	High	–
HTC compressor	Moderate	High	–
TV-1	Very low	Dominant	–
TV-2	Very low	Dominant	–
TV-3	Very low	Dominant	–
Condenser	Moderate	High	Low
Evaporator	Moderate	High	–

## Data Availability

The data presented in this study are available on request from the corresponding author.

## Abbreviations

**Nomenclature**	
$\dot{C}$	Cost flow rate ($/hr)
$\dot{E}$	Exergy flow rate (kW)
$\dot{m}$	Mass flow rate (kg/s)
$\dot{Q}$	Cooling capacity (kW)
$\dot{W}$	Work (kW)
$\dot{Z}$	Capital investment and operating and maintenance cost rate ($/hr)
A	Area (m^2^)
c	Cost per exergy unit ($/GJ)
h	Specific enthalpy (kJ/kg)
i	Interest rate (%)
n	Service life (year)
P	Pressure (kPa)
s	Specific entropy (kJ/kg/K)
T	Temperature (°C)
t	Time (hr)
U	Overall heat transfer coefficient (W/m^2^K)
y	Destruction ratio (%)
Z	Capital investment and operating and maintenance cost ($)
**Acronyms**	
CEPCI	Chemical Engineering Plant Cost Index
CFC	Chlorofluorocarbon
COP	Coefficient of Performance
CRF	Capital recovery factor
CRS	Cascade refrigeration system
GWP	Global warming potential
HC	Hydrocarbon
HFC	Hydrofluorocarbon
HTC	High temperature cycle
LMTD	Logarithmic mean temperature difference
LTC	Low temperature cycle
MTC	Medium temperature cycle
NBP	Normal boiling point, °C
ODP	Ozone depletion potential
SPECO	Specific cost of exergy
ULT	Ultra-low temperature
VCRS	Vapor compression refrigeration system
**Greek symbols**	
Δ	Difference in cascade heat exchanger
ϕ	Maintenance factor
ε	Exergy efficiency
η	Efficiency
**Subscript**	
0	Dead point
1…16	State point
CHX	Cascade heat exchanger
cold	Cold
comp	Compressor
cond	Condenser
D	Destruction
elect	Electrical
evap	Evaporator
f	Exergoeconomic factor
F	Fuel
hot	Hot
i	Individual component
in	Inlet
isen	Isentropic
L	Loss
mech	Mechanical
o	Outlet
P	Product
ref	Reference
sys	System
tot	Total
TV	Throttle valve
**Superscript**	
CI	Capital investment
O&M	Operation and maintenance
